# Investigations into a putative role for the novel BRASSIKIN pseudokinases in compatible pollen-stigma interactions in *Arabidopsis thaliana*

**DOI:** 10.1186/s12870-019-2160-9

**Published:** 2019-12-11

**Authors:** Jennifer Doucet, Hyun Kyung Lee, Nethangi Udugama, Jianfeng Xu, Baoxiu Qi, Daphne R. Goring

**Affiliations:** 1 Department of Cell & Systems Biology, University of Toronto, Toronto, M5S 3B2 Canada; 2 School of Pharmacy & Biomolecular Sciences, Liverpool John Moores University, Liverpool, L3 3AF UK; 3 College of Horticulture, Agricultural University of Hebei, Baoding City, 071001 Hebei Province China; 4 Centre for the Analysis of Genome Evolution & Function, University of Toronto, Toronto, M5S 3B2 Canada

**Keywords:** Compatible pollen, Stigma, Signaling, Pseudokinase, Receptor-like cytoplasmic kinase, Brassicaceae

## Abstract

**Background:**

In the Brassicaceae, the early stages of compatible pollen-stigma interactions are tightly controlled with early checkpoints regulating pollen adhesion, hydration and germination, and pollen tube entry into the stigmatic surface. However, the early signalling events in the stigma which trigger these compatible interactions remain unknown.

**Results:**

A set of stigma-expressed pseudokinase genes, termed *BRASSIKINs* (*BKNs*), were identified and found to be present in only core Brassicaceae genomes. In *Arabidopsis thaliana* Col-0, *BKN1* displayed stigma-specific expression while the *BKN2* gene was expressed in other tissues as well. CRISPR deletion mutations were generated for the two tandemly linked *BKNs*, and very mild hydration defects were observed for wild-type Col-0 pollen when placed on the *bkn1/2* mutant stigmas. In further analyses, the predominant transcript for the stigma-specific *BKN1* was found to have a premature stop codon in the Col-0 ecotype, but a survey of the 1001 *Arabidopsis* genomes uncovered three ecotypes that encoded a full-length BKN1 protein. Furthermore, phylogenetic analyses identified intact BKN1 orthologues in the closely related outcrossing *Arabidopsis* species, *A. lyrata* and *A. halleri*. Finally, the BKN pseudokinases were found to be plasma-membrane localized through the dual lipid modification of myristoylation and palmitoylation, and this localization would be consistent with a role in signaling complexes.

**Conclusion:**

In this study, we have characterized the novel Brassicaceae-specific family of *BKN* pseudokinase genes, and examined the function of *BKN1* and *BKN2* in the context of pollen-stigma interactions in *A. thaliana* Col-0. Additionally, premature stop codons were identified in the predicted stigma specific *BKN1* gene in a number of the 1001 *A. thaliana* ecotype genomes, and this was in contrast to the out-crossing *Arabidopsis* species which carried intact copies of *BKN1*. Thus, understanding the function of *BKN1* in other Brassicaceae species will be a key direction for future studies.

## Background

In the Brassicaceae, the early post-pollination stages of pollen adhesion and hydration, and pollen tube entry into the stigma are highly-regulated and represent the first of several stages leading to the release of the sperm cells at the ovule for fertilization (reviewed in [1–5]). The characteristic Brassicaceae “dry stigmas” lack surface secretions to facilitate pollen hydration and germination; thus, pollen recognition is required for the stigma to be receptive [6, 7]. The Brassicaceae stigma surface is covered with unicellular stigmatic papillae, and the process of pollen capture is very rapid, occurring in as little as 30 s following a compatible pollination in *Arabidopsis thaliana* [8]. Following this, the pollen coat and stigma surface components mix to form a “pollen foot” at the location of the pollen-papillar contact, and this contributes to the process of pollen adhesion [9]. The next checkpoint of pollen acceptance is pollen hydration, where the desiccated pollen grain takes up water released by the stigmatic papilla to become metabolically active [6, 10–12].

Despite being a critical step leading to successful fertilization, the cell-cell communication events that facilitate early pollen-stigma interactions are poorly understood. There are proteins in the pollen coat that are required for pollen hydration such as the *A. thaliana* GRP17 oleosin-domain protein, the EXL4 extracellular lipase, and the Pollen Coat Protein-B family (PCP-B) [13–15]. The PCP-Bs are particularly interesting as they are small cysteine-rich proteins that represent promising compatible pollen recognition factors for unknown stigma receptors. *A. thaliana pcp-bα/β/γ* triple mutants displayed impaired pollen hydration and delayed pollen tube growth on wild-type stigmas [15]. Perception of peptide ligands by receptor kinases plays a prominent role in the regulation of downstream compatible pollen-pistil interactions and pollen tube guidance, as well as the rejection of self-pollen in self-incompatible Brassicaceae species (reviewed in [1, 2, 4, 5, 16]).

Other factors identified on the pollen side for these early post-pollination stages are connected to the production of reactive oxygen species (ROS). Pollen NADPH oxidases were shown to be important for Ca^2+^-dependent ROS production in the apoplast for *A. thaliana* pollen tube elongation into the stigmatic papillar cell wall [17, 18]. ROS production was again implicated in *A. thaliana* T-DNA insertion mutants disrupting the β and γ subunits of the SNF1-related protein kinase 1 complex. Mutant *kinβγ* pollen grains displayed reduced ROS levels as a result of mitochondrial and peroxisomal defects, and this was associated with reduced hydration and germination on wild-type stigmas [19]. Finally, the *SHAKER POLLEN INWARD K*^*+*^ *channel* (*SPIK*) gene was found to be downregulated in *kinβγ* mutant pollen, and *spik* mutant pollen grains also displayed reduced hydration on wild-type stigmas [20].

On the stigmatic papillar side, ultrastructural studies of the pollen-papillar interface previously implicated both secretory activity and vacuolar expansion in the stigmatic papillae of *Brassica* and *Arabidopsis* species [21–25]. This exocyst complex, a vesicle-tethering complex composed of eight different subunits (SEC3, SEC5, SEC6, SEC8, SEC10, SEC15, EXO70 and EXO84), was implicated in mediating this secretory activity in the stigma [26–28]. Through the use of knockout mutants and stigma-specific RNA silencing constructs, all eight subunits were found to be required in the stigma for the compatible pollen acceptance. Wild-type pollen applied to stigmas from the exocyst subunit knockdown/knockout mutants displayed reduced pollen hydration and germination, and showed signs of disrupted secretion [22, 26, 27, 29, 30]. Other cellular responses in *Brassica* and *Arabidopsis* stigmatic papillae have also been connected to vesicle trafficking (reviewed in [31]). For example, *Brassica* compatible pollinations were associated with actin reorganization in the stigmatic papilla towards the pollen attachment site and microtubule depolymerization [25, 32]. Recently, another vesicle trafficking-related component, *Brassica* phospholipase Dα1, has been shown to be required in the stigma for compatible pollinations [33]. As well, changes in Ca^2+^ dynamics were observed, with small Ca^2+^ increases at the site of pollen attachment in *A. thaliana* stigmatic papillae [34]. Through transcriptome analyses of *A. thaliana* stigmas pre- and post-pollination, the ACA13 Ca^2+^ ATPase was identified as a stigmatic component and proposed to secrete Ca^2+^ for the developing pollen tube [35]. Finally, we have recently identified the secreted *Arabidopsis* E6-like 1 protein as a potential structural component of the stigmatic papillae required for these early post-pollination stages [36].

While the PCP-Bs represent potential pollen ligands for compatible pollen recognition, the corresponding recognition system in the stigma is unknown. The process of pollen acceptance by the stigma is thought to be conserved in the Brassicaceae since pollen from several Brassicaceae species were able to hydrate and germinate on *Arabidopsis* stigmas whereas pollen from non-Brassicaceae species failed to hydrate [37]. Moreover, when pollen from various species were applied to *A. thaliana* or *B. oleracea* stigmas, there was some specificity at the pollen adhesion stage [8, 38]. Thus in this reverse-genetics study, we utilized publicly available transcriptome datasets to search for potential signalling genes that display stigma-enriched expression and were conserved within the Brassicaceae. Through this approach, we identified a novel group of Brassicaceae-specific pseudokinase genes which we termed the *BRASSIKINs* (*BKNs*).

## Results

### *BKN*s are stigma-expressed receptor-like cytoplasmic kinases

**Fig. 1:**
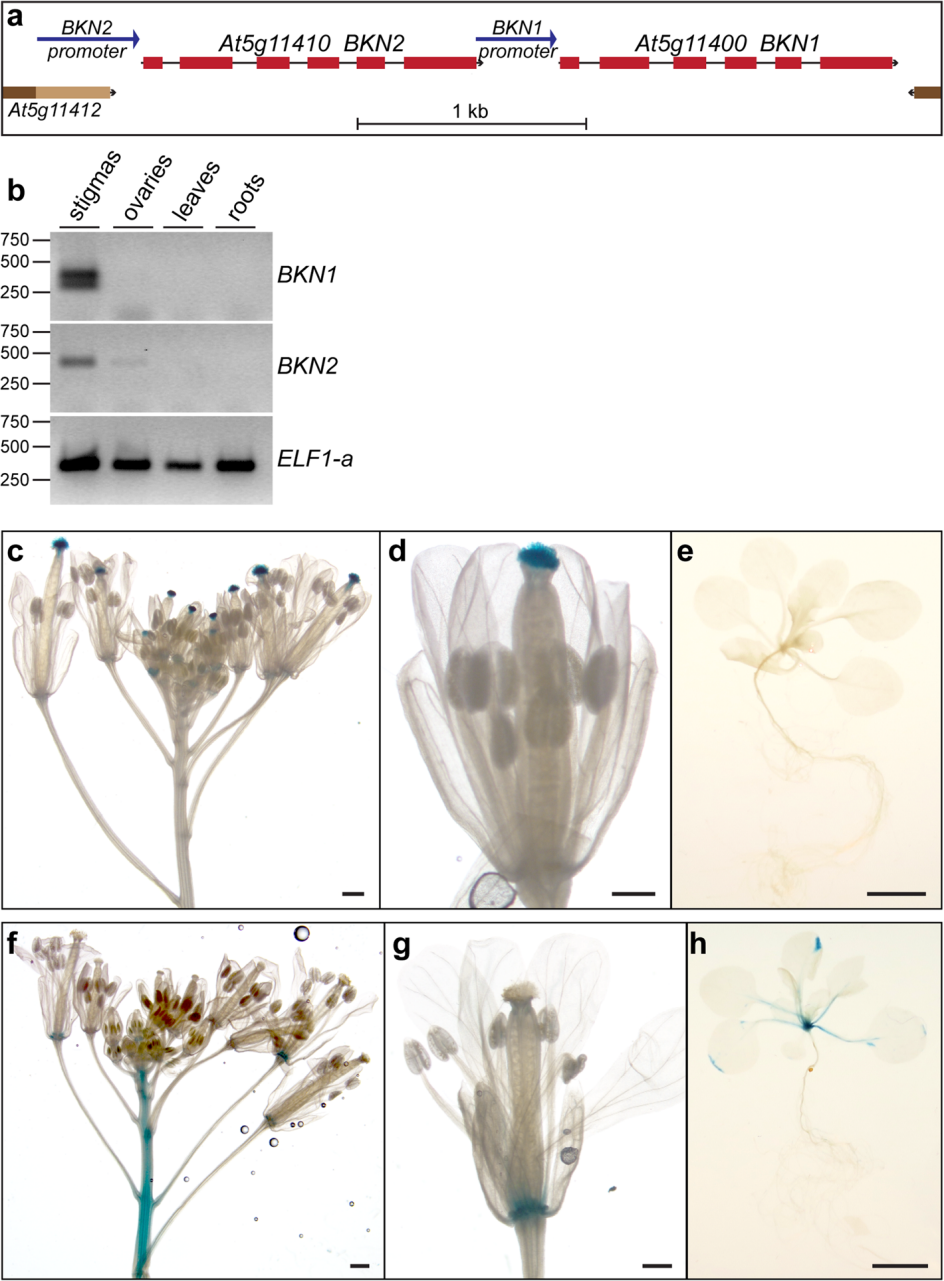
Tissue-specific expression patterns of *BKN1* and *BKN2* in *A. thaliana*. **a.** Gene structures for the tandemly linked *BKN1* and *BKN2* genes. The promoter regions used for *BKNp:GUS* constructs are shown by blue arrows. **b.** RT-PCR analysis of different tissues show *BKN1* and *BKN2* expression in the stigmas. The double PCR bands observed for *BKN1* is the result of the third intron not being properly spliced in the top band (determined by sequencing). This is also seen in the carpel RNA-Seq mapping data (Fig. 5).**c-e*.*** *GUS* staining of different tissues from *BKN1p:GUS* transgenic plants. GUS activity was specifically detected in stigmas across developmental stages in the inflorescence (c,d), and not in other tissues, including seedlings (e). **f-h.** GUS staining of different tissues from *BKN2p:GUS* transgenic plants. GUS activity was detected primarily in the floral abscission zones and stems (f, g). GUS staining was also seen in the petioles and the tips of leaves for about half of the samples (h). Scale bars are 1 cm for inflorescence images (right), and 500 μm for stage 12 flowers (centre) and 100 μm for seedlings (left)

To identifying candidate stigma signalling genes, we used the expression angler tool from the *Bio-Analytic Resource for Plant Biology* [39]. For this search, we used the stigma-specific *SLR1* gene [40] as a bait to identify other genes with similar expression patterns across the *A. thaliana* developmental series microarray datasets [36, 41, 42]. The gene, At5g11400, was a top hit (Additional file 2: Table S1) and displayed stigma-specific expression in the transcriptome datasets (Additional file 1: Figure S1, [35, 43, 44]). This gene is predicted to encode a novel receptor-like cytoplasmic kinase (RLCK) which we named *BRASSIKIN 1* (*BKN1*). Interestingly, adjacent to the *BKN1* gene is a tandemly linked paralogue, At5g11410, named *BKN2* in the *A. thaliana* genome (Fig. 1a). *BKN2* was ranked 64th in the expression angler dataset (Additional file 2: Table S1), with expression in a wider range of tissues (Additional file 1: Figure S1). Both *BKN* genes are also predicted to encode pseudokinases (discussed below) [46, 47].

The expression patterns for *BKN1* and *BKN2* were examined by RT-PCR on RNA extracted from *A. thaliana* stigmas (top ½ pistil), ovaries (bottom ½ pistil), leaves and roots. Both *BKN1* and *BKN2* were expressed in the stigma samples, with some expression for *BKN2* in ovary samples (Fig. 1b). The *BKN1* and *BKN2* expression patterns were also examined in promoter-*GUS* transgenic plants (promoter regions are indicated by arrows, Fig. 1a). For the transgenic *BKN1p:GUS A. thaliana* lines, stained inflorescences showed high levels of GUS activity in stigmas from flowers across developmental stages, but not in the other tissues in the inflorescences or in seedlings (Fig. 1c-e). The transgenic *BKN2p:GUS* inflorescences displayed GUS activity primarily in the flower abscission zones and in the stems (Fig. 1f-g). There was also GUS activity in some *BKN2p:GUS* seedlings at the leaf edges and petiole (Fig. 1h). GUS activity was not observed in *BKN2p:GUS* stigma tissues; however, this may be due to *BKN2* having much lower expression in the stigmas that may be undetectable by GUS staining (Additional file 1: Figure S1). Alternatively, the adjacent stigma specific *BKN1* promoter or other unknown regulatory regions may be responsible for the *BKN2* expression detected in the stigma tissues (Fig. 1a,b).

### Analysis of compatible pollen responses for *BKN1* and *BKN2* single and double knockout mutants in *A. thaliana*

**Fig. 2:**
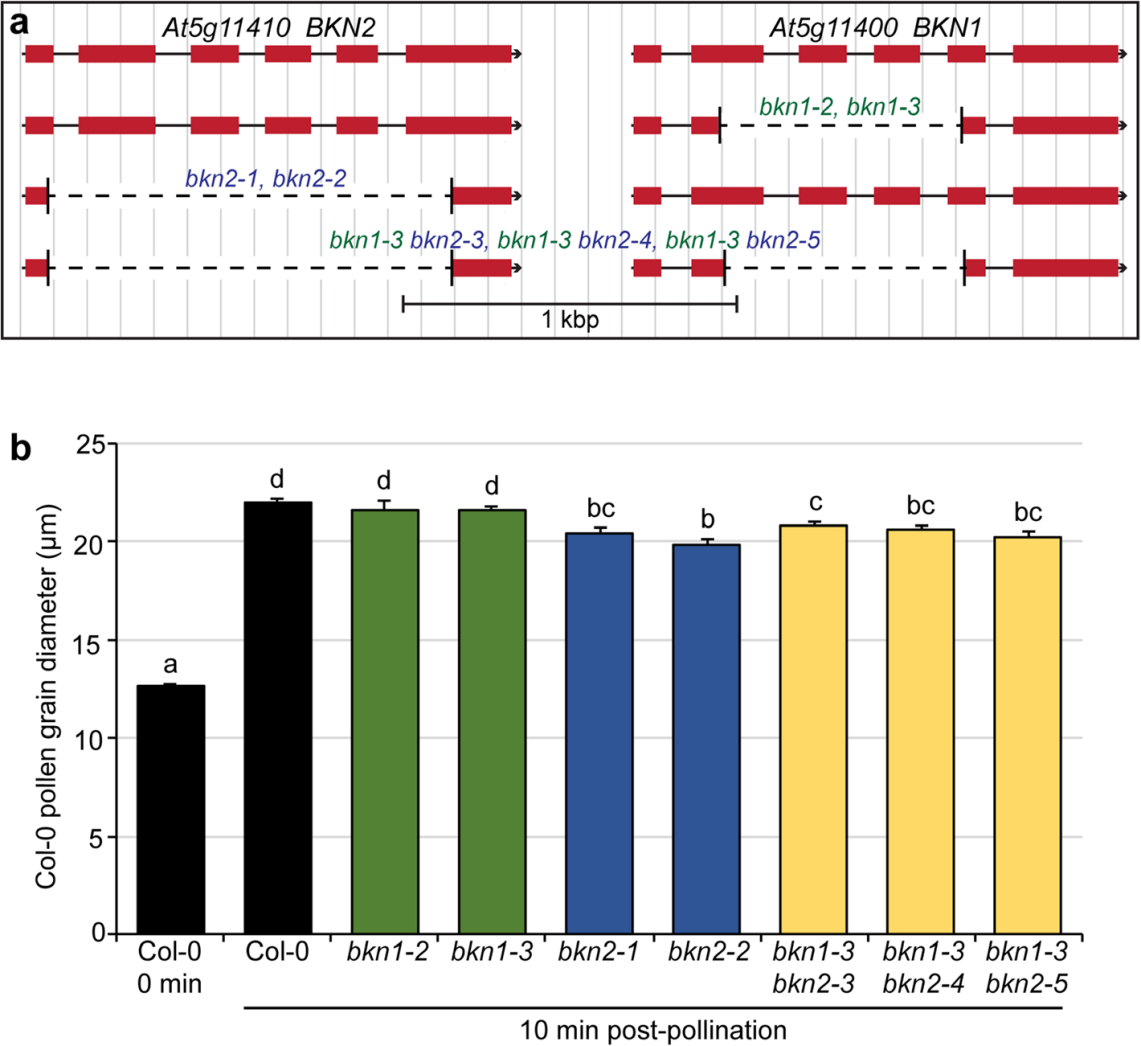
Pollen hydration assays for *A. thaliana* Col-0 *bkn1* and *bkn2* CRISPR deletion mutants. **a.** Structure of the tandemly linked *BKN1* and *BKN2* genomic region depicting the locations of CRISPR deletions. The *bkn1* and *bkn2* deletion mutants were generated separately and the CRISPR-generated deletion were confirmed by sequencing. The *bkn1–3* mutant was then transformed with a CRISPR construct to delete the *BKN2* gene resulting in the *bkn1 bkn2* double mutants. **b.** Pollen hydration assays at 10 min post-pollination. Wild-type Col-0 pollen was applied to Col-0 stigmas and the different *bkn* mutant stigmas and left for 10 min. Pollen hydration results in a change in pollen grain diameter which was measured at 10 min post-pollination. Stigmas carrying a mutation in the *BKN2* gene showed reduced Col-0 pollen hydration compared to Col-0 stigmas. *n* = 30 pollen grains per line. Letters represent statistically significant groupings of *p* < 0.05 based on a one-way ANOVA with a Duncan post-hoc test

Given *BKN1*’s stigma-specific expression, we investigated whether *BKN1* was required for compatible pollinations by examining loss-of function mutants. A knockout line with a T-DNA inserted in the fifth exon of *BKN1* was assessed for post-pollination responses (*bkn1–1*; Additional file 1: Figure S2a, b). The *bkn1–1* mutant plants did not display any discernible developmental defects and appeared fully fertile with wild-type siliques. Furthermore, pollinated *bkn1–1* pistils stained with aniline blue were similar to wild-type for adhered pollen grains and pollen tube growth (Additional file 1: Figure S2c, d). Given that this mutant displayed some expression upstream of the T-DNA insertion, additional *BKN1* mutants were generated using a CRISPR/Cas9 genome editing system [48]. Furthermore, a similar approach was taken for BKN2 since it could potentially function redundantly to BKN1. Single deletion mutants were generated resulting in two new independent homozygous mutants for each *BKN1* and *BKN2*: *bkn1–2* and *bkn1–3*, and *bkn2–1* and *bkn2–2* (Fig. 2a).

**Fig. 3:**
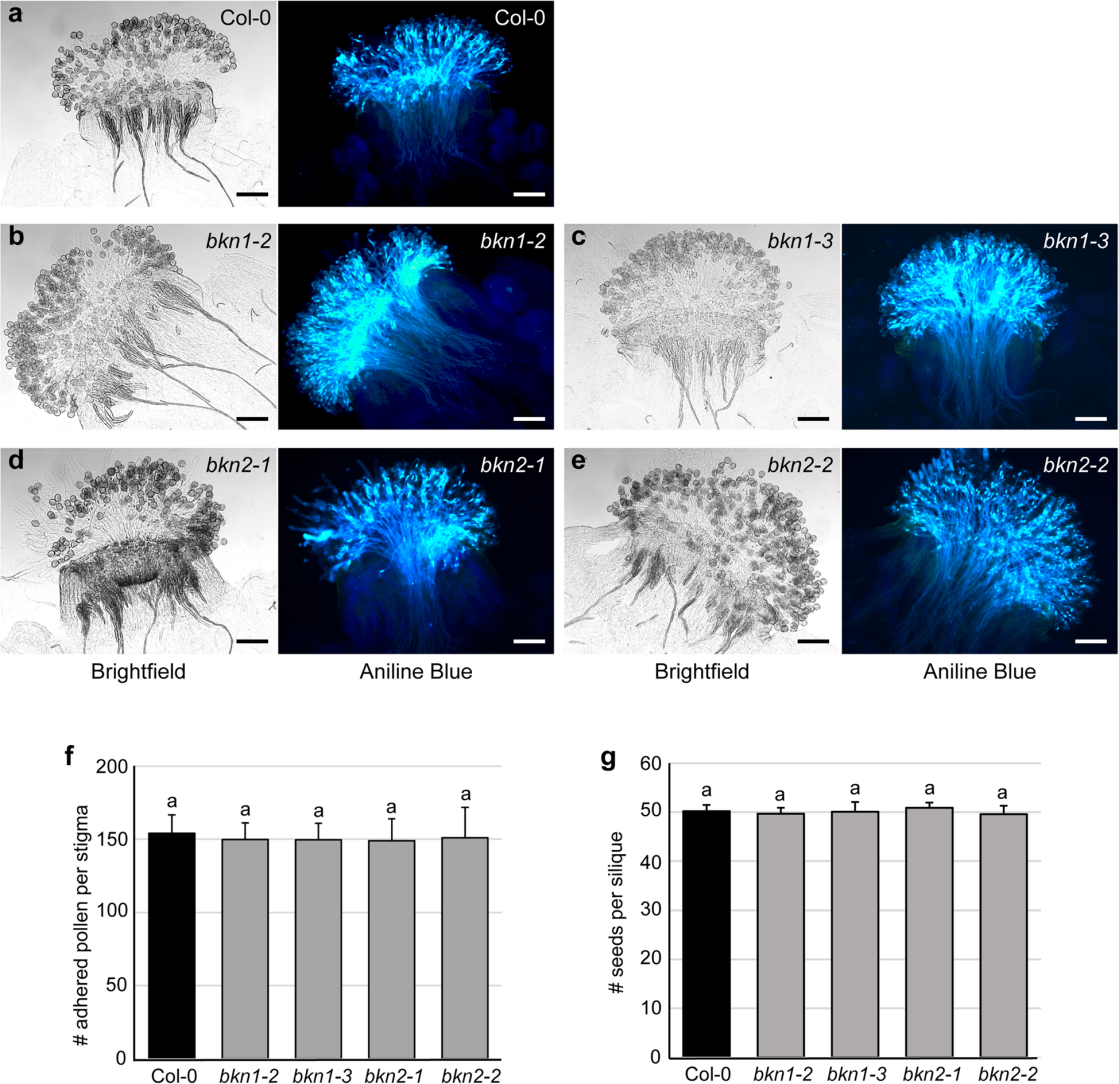
Analysis of the single *bkn1* and *bkn2* CRISPR mutants. **a-e**. Stage 13 pistils were pollinated with wild-type Col-0 pollen for 2 h and then fixed and stained with aniline blue. Brightfield images (left) show pollen grains adhered to the stigmatic papillae, and fluorescent images (right) show aniline blue stains of callose deposits in the pollen tubes. Representative images of pollinated Col-0 pistils (a) *bkn1–2* (b) *bkn1–3* (c), *bkn2–1* (d) and *bkn2–3* (e) show similar pollen adhesion and pollen tube penetration. Scale bar = 100 μm. **f.** Number of Col-0 pollen grains adhered to stigmas following aniline blue staining at 2-h post-pollination for Col-0 and the *bkn1* and *bkn2* mutants. During this staining procedure, ungerminated pollen grains are washed away and so the number of adhered pollen grains can be quantified. *n* = 10 stigmas per line. **g.** Seed set rates, following hand-pollination with Col-0 pollen. All *bkn1* or *bkn2* mutants display normal seed set, similar to Col-0. n = 10 siliques per line. Letters represent statistically significant groupings of p < 0.05 based on a one-way ANOVA with a Duncan post-hoc test. No significant differences were observed for (f) and (g)

Similar to *bkn1–1*, all four CRISPR deletion mutants *bkn1–2*, *bkn1–3*, *bkn2–1* and *bkn2–2* displayed wild-type pollen tube growth in aniline blue stained pistils that had been manually pollinated with wild-type Col-0 pollen (Fig. 3a-e). There were no discernable phenotypes at this stage for individual *bkn1* and *bkn2* loss-of-function mutants. These mutants also did not show any observable defects in the number of pollen grains adhered to the stigma or seed set, relative to Col-0 (Fig. 3f-g). We then examined one of the earliest post-compatible pollination stages, pollen hydration, which is dependent on water release from the stigma [6, 26, 27]. Col-0 pollen was applied to all stigmas and pollen hydration was assessed by measuring the diameter of pollen grains which become rounder in shape with water uptake. Col-0 pollen on Col-0 stigmas had a mean pollen grain diameter of 21.9 μm at 10 min post-pollination compared to 12.6 μm at 0 min. The *bkn1–2* and *bkn1–3* mutant stigmas supported similar levels of Col-0 pollen hydration when compared to Col-0 stigmas (Fig. 2b). In contrast, Col-0 pollen place on the *bkn2–1* and *bkn2–2* mutant stigmas showed a small but significant decrease in diameter at 10 min post-pollination. This suggested that there was a mild Col-0 pollen hydration defect on the *bkn2–1* and *bkn2–2* mutant stigmas (Fig. 2b).

**Fig. 4:**
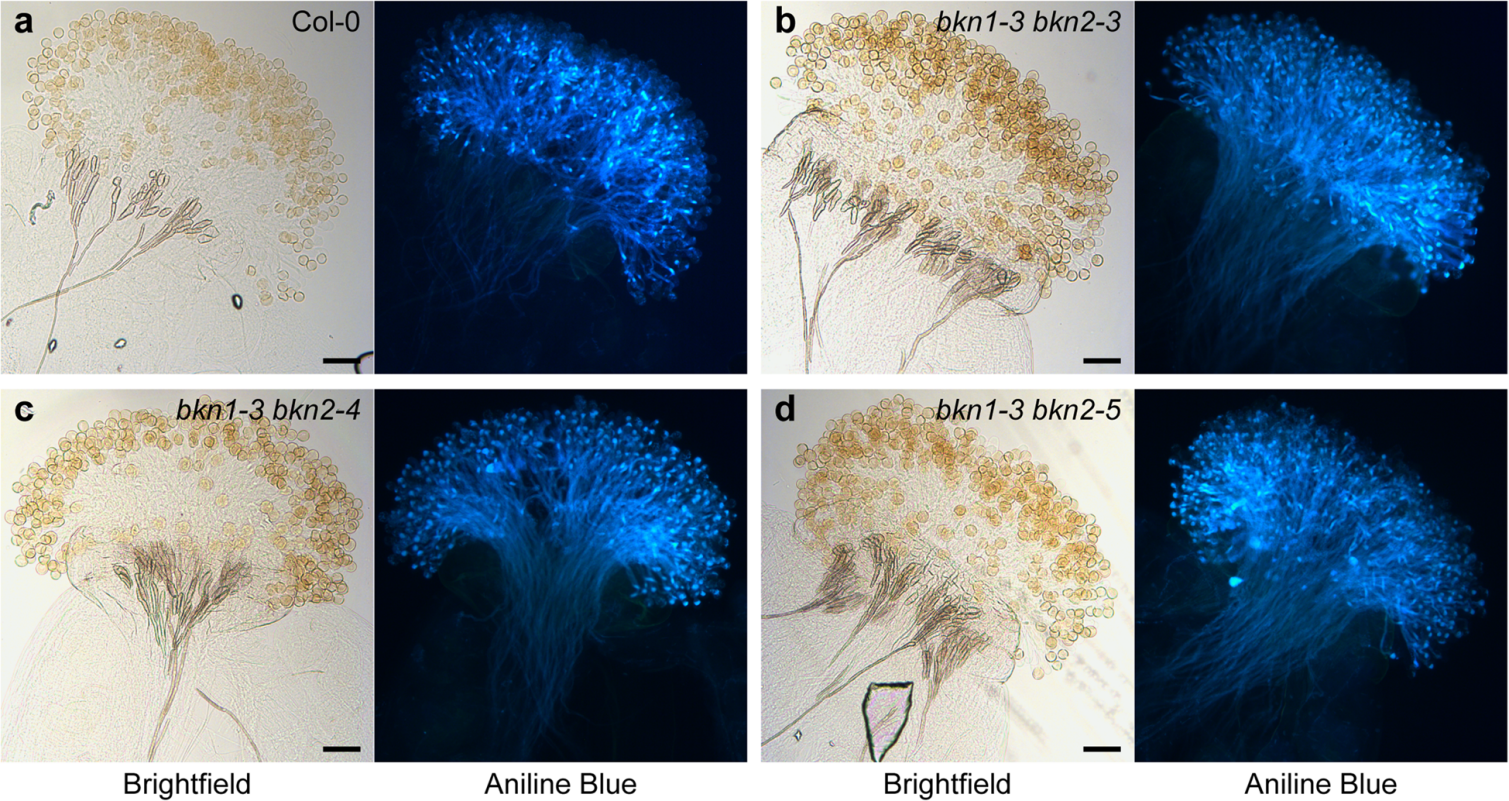
Representative images of aniline blue-stained stigmas from the *bkn1 bkn2* mutants. **a-d.** Aniline blue-stained pistils, following 2 h pollination with Col-0 pollen. Brightfield images (left) show pollen grains adhered to the stigmatic papillae, and fluorescent images (right) show aniline blue-stained pollen tubes (a), *bkn1–3 bkn2–3* (b), *bkn1–3 bkn2–4* (c) and *bkn1–3 bkn2–5* (d). All show similar levels of pollen adhesion and pollen tube growth. Scale bar = 100 μm

To test for potential functional redundancy, double *bkn1-bkn2* mutants were generated. Since the *BKN1* and *BKN2* genes are tandemly arrayed, the strategy taken was to transform a transgene-free *bkn1–3* mutant with a *BKN2* CRISPR construct to knock out both genes. From this screen, three new *bkn2* mutants, *bkn2–3*, *bkn2–4* and *bkn2–5*, were identified in the *bkn1–3* background (Fig. 2a). Pollen hydration assays were conducted on these double *bkn1-bkn2* homozygous mutants, and again mild, but significant reductions were observed for the Col-0 pollen placed on these mutant stigmas (Fig. 2b). Similar to the single mutants, pollinated double *bkn1-bkn2* mutant pistils stained with aniline blue displayed wild-type levels of adhered pollen grains and pollen tube growth (Fig. 4). Thus, there did not appear to be any additive effect by knocking out both *BKN1* and *BKN2*, and suggests that the highly expressed, stigma-specific *BKN1* did not display a noticeable function in pollen-stigma interactions.

### Variations in predicted protein translation products for *BKN1* in different *A. thaliana* ecotypes

**Fig. 5:**
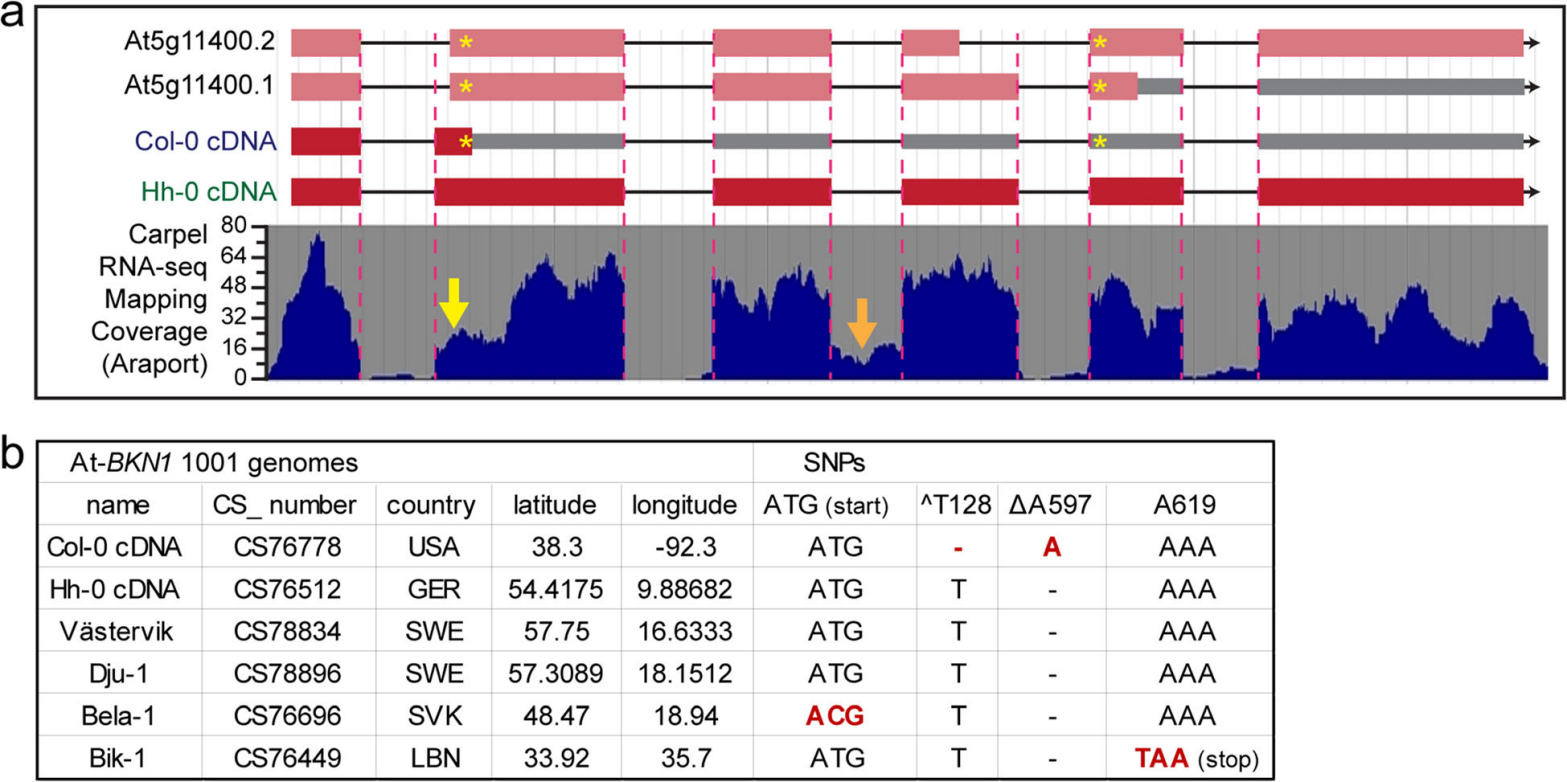
*A. thaliana BKN1* gene models and ecotype polymorphisms. **a.** *A. thaliana BKN1* gene models are shown with carpel RNA-Seq mapping coverage from Araport [45]. Yellow astericks (*) mark two in/del SNPs in Col-0 *BKN1* when compared to Hh-0 *BKN1* and *A. lyrata BKN1*. For the BNK1 Col-0 gene annotations and cDNA, the first asterisk marks a 1 bp deletion (T) resulting in an adjacent premature stop codon and the second asterisk marks a 1 bp insertion (A) that would result in a downstream premature stop codon for the At5g11400.1 annotation. Based on the reduced carpel RNA-Seq coverage at the 5′ end of the *BKN1* exon 2, there may be alternate splice sites in use (yellow arrow) and some of these potential alternate splice junctions would restore the *BKN1* Col-0 reading frame to produce a longer protein as predicted for the At5g11400.1 and At5g11400.2 annotations. See also Additional file 1: Figure S3 and S4. The orange arrow delineates the third intron that is not properly spliced in the top RT-PCR band in Fig. 1b. **b.** *A. thaliana BKN1* polymorphisms in different ecotypes. In addition to Hh-0, Västervik and Dju-1 are predicted to encode a full length BKN1 protein (based on genomic sequencing). Bela-1 and Bik-1 displayed other SNPs that disrupt the BKN1 open reading frame (see also Additional file 1: Figure S6)

As the *bkn1* mutants displayed wild-type post-pollination phenotypes, the predicted protein sequences encoded by the *BKN* genes were examined more closely. *BKN* cDNAs were cloned from the *A. thaliana* Col-0 ecotype and compared to the TAIR/Araport gene annotations [45, 49]. While the *BKN2* cDNA sequence and predicted amino acid sequence matched the gene annotation, the full-length *BKN1* cDNA showed some differences (Fig. 5a, Additional file 1: Figure S3). Importantly, the second exon in the cDNA included an additional 17 bp at the 5′ end resulting in a frameshift and a premature stop codon (Fig. 5a, asterisk). As a result, the predicted *A. thaliana* Col-0 BKN1 protein would only be 42 aa in length, in comparison to the predicted 304 aa (Additional file 1: Figure S4). The cloned BKN1 cDNA matched the carpel RNA-Seq mapping data displayed on Araport (Fig. 5a); nevertheless, there also appeared to be potential alternative splice sites at the beginning of the second exon that could restore the BKN1 reading frame and encode a larger protein (i.e. the *BKN1* gene annotations; yellow arrow in Fig. 5a; Additional file 1: Figure S3, S4). While two *BKN1* RT-PCR bands were observed in Fig. 1b, the larger band was identified by sequencing to include the third intron, rather than an alternatively spliced transcript. Signs of the unspliced third intron were also present in the carpel RNA-Seq mapping displayed on Araport (orange arrow in Fig. 5a). Despite several attempts, we were unable to clone *BKN1* cDNAs that corresponded to the TAIR/Araport gene annotations.

A search for *BKN* orthologues in the genomes of two outcrossing *Arabidopsis* species, *A. lyrata*, and *A. halleri*, uncovered *BKN1* coding regions that were predicted to be fully intact. This was confirmed by cloning the corresponding cDNAs from *A. lyrata* (Additional file 1: Figure S3, S4 and S5). When the *BKN1* sequences were aligned, two indels were identified in the *A. thaliana* Col-0 *BKN1* cDNA sequence that would disrupt the reading frame (Fig. 5, asterisks), the first being a 1 bp deletion (ΔT128) and the second being a 1 bp insertion (^A597; Additional file 1: Figure S3). We then searched through the 1135 genomes dataset to determine how widespread these *BKN1* indels were across the different *A. thaliana* ecotypes [50]. Most ecotypes carried ΔT128 causing the premature stop codon in Col-0 *BKN1* (Additional file 3: Table S2). Interestingly, three *A. thaliana* ecotypes were predicted to have fully intact *BKN1* coding regions. The first ecotype identified was Hh-0, and the corresponding cDNA was cloned and confirmed by sequencing to encode a full-length BKN1 protein, similar to Al-BKN1 (Fig. 5, Additional file 1: Figure S3, S4 and S5). Subsequent searches identified two other ecotypes, Dju-1 and Västervik, that were also confirmed to carry the same two indels as Hh-0 to encode a full-length BKN1 protein (Fig. 5b, Additional file 1: Figure S6, Additional file 3: Table S2). However, in a number of other ecotypes, the presence of the two ORF-restoring indels (^T128, ΔA597) were associated with new SNPs that would again knock out the *BKN1* coding region. This included the loss of the start methionine (ATG → ACG) and a new stop codon (TAA) downstream of ΔA597 (Fig. 5, Additional file 1: Figure S6, Additional file 3: Table S2). With Hh-0 expressing an intact At-BKN1 gene, pollen hydration assays were conducted on Hh-0 flowers to see if there was any variation at this early post-pollination stage and then compared to Col-0 in reciprocal pollinations, but no obvious differences were observed (Additional file 1: Figure S7).

### BKNs are conserved within the Brassicaceae but are absent in species outside this family

Given the *BKN1* polymorphisms found in the *Arabidopsis* species genomes, we also investigated related *BKN* genes in other plant species. The BKNs are part of the group VII RLCKs (Additional file 1: Figure S8; as defined by [51]) which include a number of important signalling proteins such as the BOTRYTIS-INDUCED KINASE1 (BIK1 [52]) and the various PBS1-Like (PBL) proteins associated with plant immune signalling [53, 54]. The BKNs are most closely related to CASTAWAY (CST [55]) and PBL31 [53] (Additional file 1: Figure S8). RLCKs are related to plant receptor kinases [51, 56], except that they lack extracellular domains and typically function in complexes with receptor kinases [57–59]. Alignments between BKN1 and BKN2 with CST (an active kinase involved in floral abscission [55]) clearly show that the BKNs are missing several key residues for ATP binding and catalytic activity, including the glycine-rich loop and the VAIK, HRD and DFG motifs, and as a result, are defined as being pseudokinases [46] (Additional file 1: Figure S9). Although some pseudokinases may exhibit partial kinase activity, BKNs are predicted to be inactive due to the number of missing residues, particularly the glycine-rich loop and the VAIK motif, which are required for catalytic activity [46, 60].

**Fig. 6:**
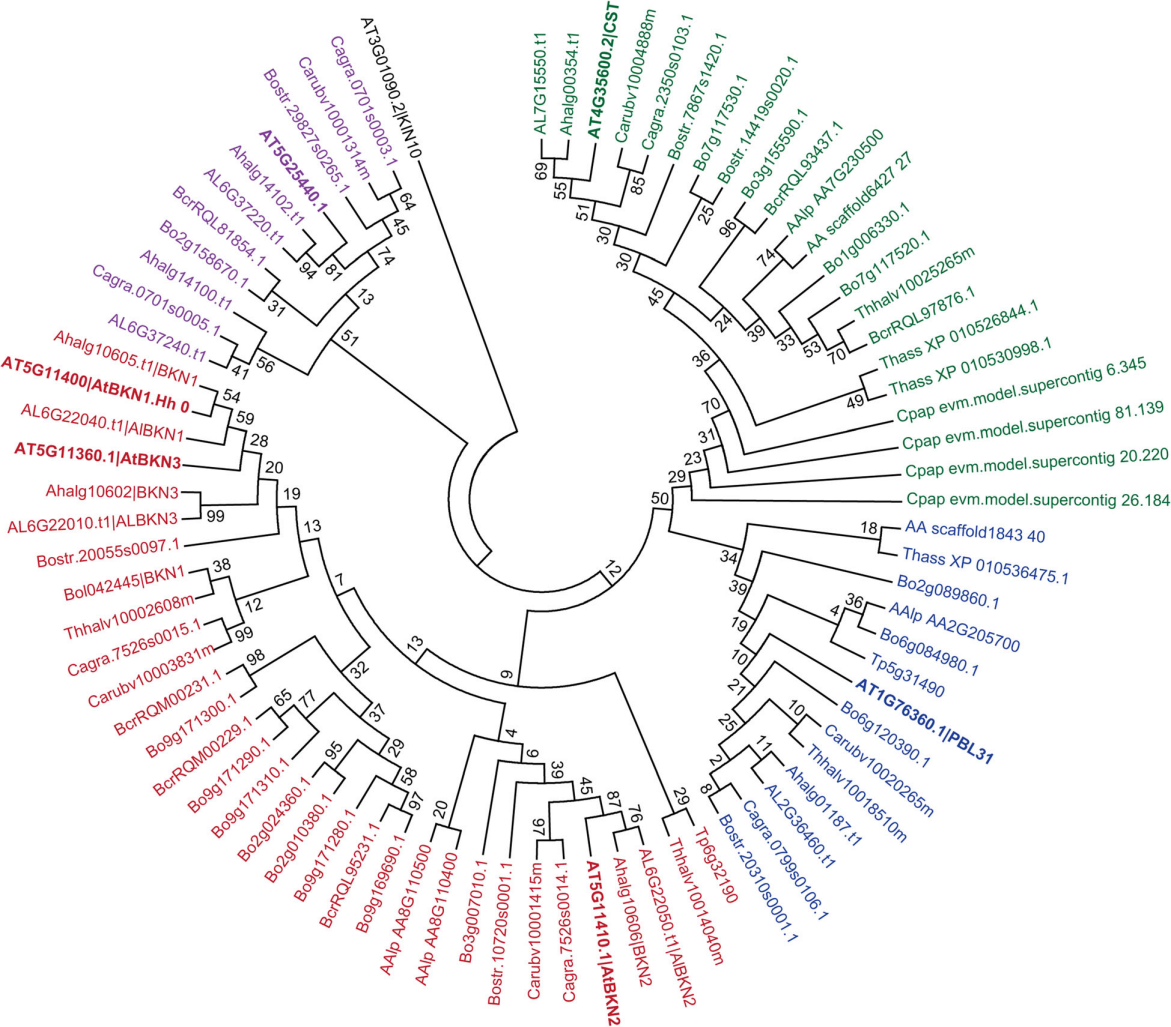
Phylogenetic analysis of Brassicaceae BKN and closely-related RLCK group VIIa protein sequences. Sequences are from *Arabidopsis thaliana, A. lyrata, A. halleri, Arabis alpina, Boechera stricta, Brassica cretica, B. oleracea*, *Capsella rubella, C. grandiflora, Eutrema salsugineum* (formerly *T. halophila*), *Schrenkiella parvula* (formerly *T. parvula*), *Aethionema arabicum, Tarenaya hassleriana*, and *Carica papaya*. Red branches represent the BKN clade, Magenta branches represent the At5g25440-related RLCKs, Blue branches represent the At1g76360-related RLCKs, Green branches represent the CST (At4g35600)-related RLCKs. *A. thaliana* protein sequences for At5g25440.1, At1g76360.1 and At4g35600.2 were selected for searches and phylogenetic analyses as they clustered with the BKNs in the RLCK-VIIa tree (Additional file 1: Figure S7). KIN10 (At3g01090.2, SnRK1 kinase) was chosen as an outgroup. The analysis involved 90 amino acid sequences and the sequences were aligned using ClustalW [62] in the MEGA 7 software [63]. The N-and C-terminal ends of the alignment were trimmed (see Supplemental files for sequences and alignment), and the tree was constructed using the Maximum Likelihood method [64] in the MEGA 7 software. All positions containing gaps and missing data were eliminated, and a total of 28 positions was in the final dataset. The tree generated in MEGA 7 represents the bootstrap consensus tree inferred from 1000 replicates [65]

To investigate the distribution of *BKN* genes in plant genomes, BLAST searches were conducted using the *A. thaliana* BKN amino acid sequences along with three closely related RLCK amino acid sequences: At5g25440, PBL31 (At1g76360) and CST (At4g35600). These three predicted proteins were selected as they cluster with the BKNs in the RLCK-VII tree (Additional file 1: Figure S8). In these searches, a third *A. thaliana BKN* paralogue was located nearby on chromosome 5, At5g11360 (*BKN3;* Additional file 1: Figure S4). At-*BKN3* is predicted to have a large internal deletion of ~ 140 amino acids, while the corresponding orthologues in *A. lyrata* and *A. halleri* encode a full length BKN3s (Additional file 1: Figure S4 and S5). We identified BKN homologues for all Brassicaceae genomes searched, including *A. lyrata, A. halleri, Arabis alpina, Boechera stricta, Capsella rubella, C. grandiflora, Brassica cretica, B. oleracea, Eutrema salsugineum* (formerly *Thellungiella halophila*) and *Schrenkiella parvula* (formerly *T. parvula*). The number of homologues ranged from one in *S. parvula* to nine in *B. oleracea*. Interestingly, the BKNs were all predicted to be the pseudokinases (Additional file 1: Figure S9) and only found in core Brassicaceae genomes, not in the genome of the basal Brassicaceae species, *Aethionema arabicum* [61]. Our searches were expanded to include two genomes from other Brassicales families (*Tarenaya hassleriana*, Cleomaceae; *Carica papaya*, Caricaceae), and a phylogenetic analysis of the retrieved sequences indicated an absence of *BKN* homologues in these genomes as well (Fig. 6). All of the core Brassicaceae BKNs were found in a clade that was distinct from the homologues for the other group VIIa RLCKs (At5g25440, CST, PBL31; Fig. 6).

### Plasma membrane localization of BKNs by predicted N-terminal myristoylation and palmitoylation sites

**Fig. 7:**
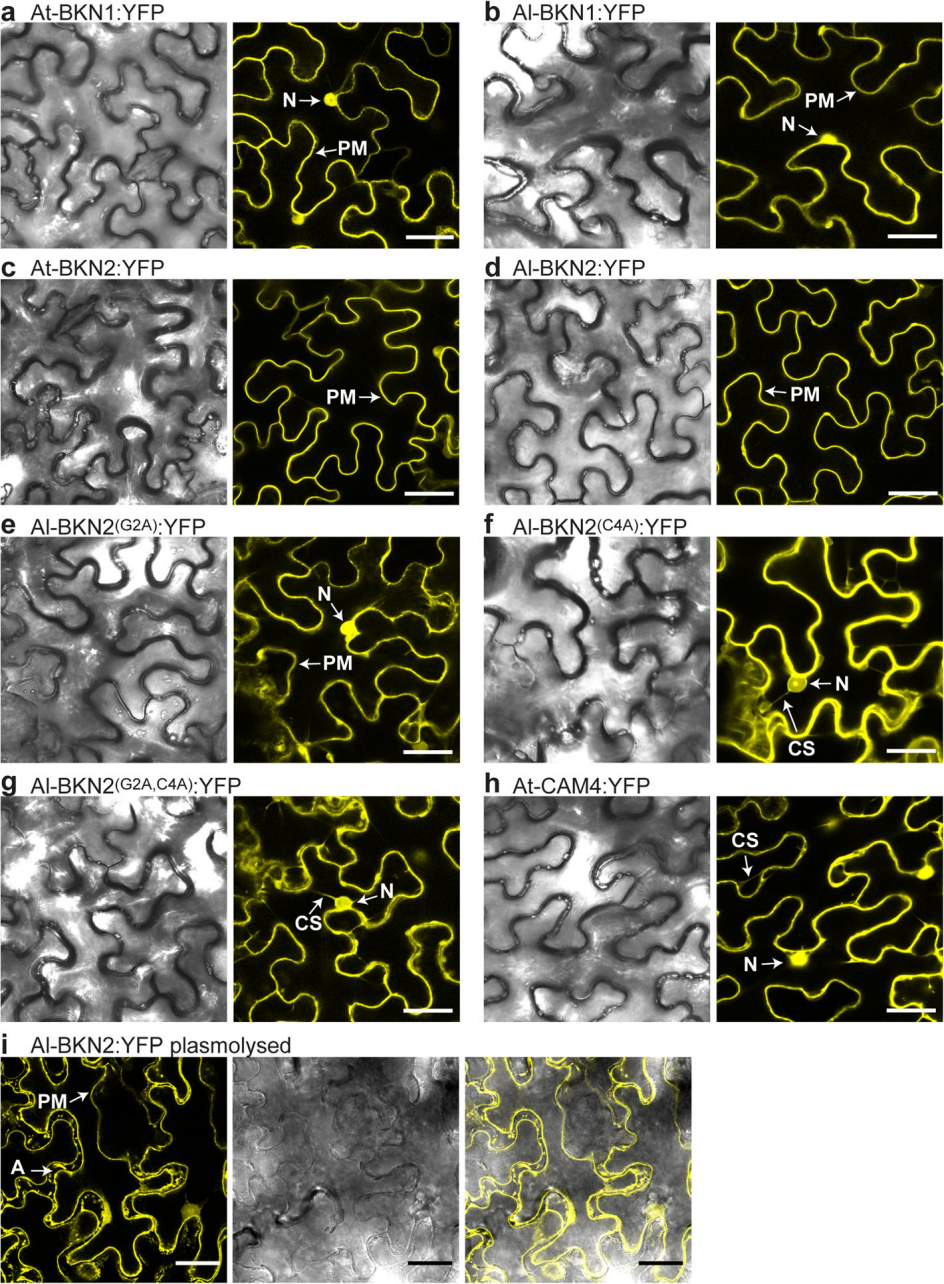
Confocal microscopy imaging of *N. benthamiana* leaves infiltrated with C-terminal BKN:YFP fusion proteins. **a-b.** BKN1 is predominantly localized to the plasma membrane (PM) with some nuclear localization (N) for both At-BKN1 (a) and Al-BKN1 (b). The BKN1 cDNA from the Hh-0 ecotype was used for expressing the At-BKN1 protein (see text). **c-d.** BKN2 is predominantly localized to the plasma membrane (PM) for both At-BKN2 (c) and Al-BKN1 (d).**e-g.** Plasma membrane localization is disrupted for Al-BKN2 versions mutated at the myristoylation (G2A) and palmitoylation (C4A) sites. Images are shown for the single G2A mutant (e), single C4A mutant (f) and double G2A C4A mutant (g) versions of Al-BKN2. Localization is seen in the cytoplasm (CS = cytoplasmic strands) and nucleus (N). **h.** *A. thaliana* CAM4:YFP was used to compare the localization of YFP fluorescence in the cytoplasm (CS = cytoplasmic strands) and nucleus (N). **i.** Cells infiltrated with Al-BKN2 were plasmolysed using 0.8 M mannitol. Localization in plasmolysed cells remains predominantly at the cell periphery. There is also some localization to Hechtian strands in the apoplastic space (A). Hechtian strands are plasma membrane tubules connected to the cell wall [80]. YFP fusion constructs were infiltrated at OD600 = 0.5, and images were taken 24 h post-inoculation. Scale bars = 30 μm

Despite lacking kinase activity, pseudokinases do play a variety of roles in biological systems and typically are in association with other signalling proteins at the cell membrane [66–74]. While the BKNs, as typical RLCKs, lack extracellular and transmembrane domains, they have conserved residues for N-terminal myristoylation and/or palmitoylation. These N-terminal lipid anchors can target proteins to the cell membrane where they would be proximal to other signalling proteins and receptors [75–77]. The presence of a glycine at position two is essential for myristoylation, while the cysteine at position 4 is required for palmitoylation [78]. Interestingly, all the Brassicaceae BKN homologues have a predicted myristoylation site (G2, Additional file 1: Figure S9) while several also have a predicted palmitoylation site at the N-terminus (C4; Additional file 1: Figure S9). Specifically, *A. thaliana* BKN1, *A. thaliana* BKN2 and *A. lyrata* BKN2 have both the G2 and C4 sites while *A. lyrata* BKN1 only has the G2 myristoylation site (Additional file 1: Figure S5 and S9). Using a transient expression system in *Nicotiana benthamiana* leaf epidermal cells, C-terminal YFP fusions [79] of the four proteins were then tested for potential plasma membrane localization. Full-length At-BKN1 from Hh-0 was tested along with At-BKN2 from Col-0, Al-BKN1 and Al-BKN2 (Fig. 7). All four BKN:YFP proteins appeared to be predominantly localized to the plasma membrane (Fig. 7a-d), with At-BKN1:YFP and Al-BKN1:YFP also showing some localization to the nucleus (Fig. 7a-b). It was unclear whether the unexpected partial nuclear localization is related to the protein function or an artifact caused by cleavage and mis-localization of the YFP. As well, Al-BKN1:YFP’s pattern of localization to the plasma membrane did not appear to be as tight as the other BKNs, but this may be related to Al-BKN1 only having a myristoylation site [81]. Myristoylation allows for transient associations with the membrane while the combination of palmitoylation and myristoylation more effectively anchors proteins to the plasma membrane, though these protein modifications remain reversible to facilitate transient membrane associations [81].

**Fig. 8:**
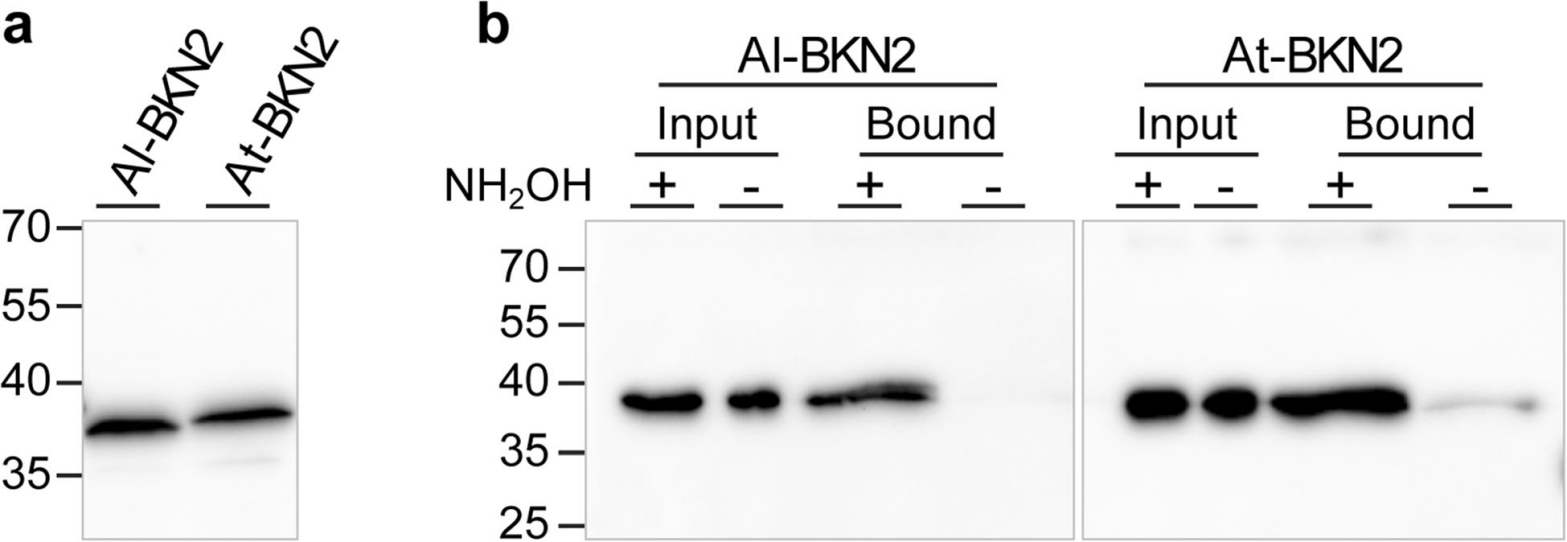
Palmitoylation of Al-BKN2 and At-BKN2. (**a**) Protein expression and (**b**) Palmitoylation assay for Al-BKN2 and At-BKN2. BKN2 proteins were purified from yeast cells and tested for *S*-palmitoylation using the in vitro acyl-RAC assay [82]. The BKN2 proteins were detected via C-terminal V5 epitope tags and western blotting with an anti-V5 antibody. The presence of a band in the + NH_2_OH bound lanes indicates that both BKN2 proteins were palmitoylated when expressed in yeast cells

With Al-BKN2:YFP and At-BKN2:YFP containing both the G2 and C4 sites and showing strong plasma membrane localization, we also tested if these proteins could be palmitoylated when expressed in yeast cells. The BKN2 proteins were isolated from transformed yeast cells (Fig. 8a), and then tested for *S*-palmitoylation using the in vitro acyl-RAC (resin-assisted capture) assay [82]. After free thiols were blocked, the proteins were either treated with hydroxylamine (+) to remove palmitoylate and exposing free thiols at the palmitoylation sites or left untreated (−). Both Al-BKN2 and At-BKN2 were detected in the (+) lanes indicating that these proteins had been S-palmitoylated and were captured on the thiol-reactive resin following hydroxylamine treatment (Fig. 8b). Finally, since Al-BKN2:YFP displayed particularly high florescent levels as well as strong localization to the plasma membrane (Fig. 7d), we tested the effects of disrupting the myristoylation and palmitoylation sites on its localization pattern. Amino acid substitutions of the myristoylation (G2A) site, the palmitoylation (C4A) site or both (G2A, C4A) in Al-BKN2:YFP disrupted its plasma membrane localization, resulting in mis-localization to the nucleus and the cytoplasm of *N. benthamiana* leaf epidermal cells (Fig. 7e-g), similar to the CAM4:YFP control (Fig. 7h). The cells infiltrated with *Al-BKN2:YFP* were also plasmolysed by treating with 0.8 M mannitol to cause cell shrinkage and plasma membrane dissociation from the cell wall. BKN2:YFP localization was observed at the plasma membrane, at the sites where the plasma membrane has detached from the cell wall, and with Hechtian strands in the apoplastic space (Fig. 7i). Similar results were observed for the other BKNs following plasmolysis (Additional file 1: Figure S10). With the Al-BKN2 versions mutated at the myristoylation (G2A) and/or palmitoylation (C4A) sites, disrupted plasma membrane localization was again observed in the plasmolysed cells with the YFP signal becoming more diffuse and some localization occurring in the nucleus (Additional file 1: Figure S10). Thus, this data strongly supports that the BKNs have N-terminal lipid anchors to localize to the plasma membrane, and disruption of the fatty acid modification sites (G2, C4) causes a mis-localization to other subcellular compartments.

## Discussion

In this study, we have investigated a novel family of pseudokinase genes, the *BRASSIKINs* (*BKNs*) that are only found in core-Brassicaceae species. The Brassicaceae genomes examined typically carried two to three different *BKN* genes, except for the *Brassica* genomes which tended to have higher numbers, up to nine predicted *BKN* genes for the diploid species. BKNs belong to the receptor-like cytoplasmic kinase (RLCK) subfamily, and as such lack an extracellular domain required for external signal perception. RLCKs play diverse roles in plant cell signalling often through interactions with receptor kinases [57, 58], and so it is conceivable that BKNs function in a receptor kinase complexes for signaling pathways. For instance, *A. thaliana* CASTAWAY (CST) is a plasma membrane localized RLCK that has been shown to interact with two receptor kinases, HAESA and EVERSHED, and function as an inhibitor of floral organ abscission [55]. CST also localized to the plasma membrane through N-terminal myristoylation and palmitoylation sites, and this localization pattern was shifted towards the cytoplasm when the N-terminal lipid anchor sites were mutated [55]. Here, we have shown that BKN1 and BKN2 have predicted N-terminal palmitoylation and/or myristoylation sites and localized to the plasma membrane in *N. benthamiana* epidermal cells. As well, yeast-expressed BKN2 was confirmed to be palmitoylated using the in vitro acyl-RAC assay. Finally, we observed that mutations of the myristoylation and palmitoylation sites for Al-BKN2 disrupted membrane localization in *N. benthamiana* epidermal cells. Thus, the plasma membrane localization of BKN1 and BKN2 could position these RLCKs for interactions with receptor complexes.

While many RLCKs, such as CST, are functional kinases, the BKNs are predicted to be pseudokinases meaning that they lack the catalytic motifs required for phosphotransfer [46, 60]. There are examples of pseudokinases with very low levels of autophosphorylation activity and this typically requires the catalytic lysine of the VAIK motif, and usually the aspartates of the HRD and DFG motifs as well [46]. In addition, while most pseudokinases are devoid of catalytic activity, it is estimated that close to 40% of all pseudokinases are capable of nucleotide binding [46]. However, the BKNs lack all of the key motifs required for ATP binding and catalytic activity, including VAIK with the key catalytic lysine as well as the HRD and DFG motifs [46, 60]. In a recent study, 168 of the 1005 *Arabidopsis* predicted kinases were classified as pseudokinases [47]. A number of these *Arabidopsis* pseudokinases (also referred to as atypical kinases [83]) have been shown to have biological functions related to signalling [66–74, 84, 85]. One example is the Brassinosteroid Signalling Kinases (BSKs), which are a group of 12 closely-related, functionally redundant cytoplasmic pseudokinases involved in brassinosteroid signalling through interactions with the receptor BRI1 [66, 67, 86]. The BSKs are phosphorylated by the BRI1 receptor kinase during BR hormone perception and are proposed to function as scaffolds in the signaling complex [67, 87]. Another example is the stem cell signalling protein CORYNE (CRN) which interacts with the receptor CLAVATA 2 to promote localization to the plasma membrane [88] and functions with receptor kinases to regulate stem cell fate in the shoot and root apical meristems [68, 69, 89]. Finally, roles for pseudokinases have been defined in immune complexes that regulate plant immunity responses [70, 84, 85, 90]. Interestingly, Liu et al. [91] recently discovered a role for BKN2 in plant immunity (SUPPRESSOR OF ZED1-D2; SZE2) and found that it was localized to the plasma membrane as part of an immune complex. Thus, given the tendency of pseudokinases to function in protein complexes, potential interactors will need to be identified in the stigma to further understand the functions of BKNs.

Our interest in the BKN family started with a search for stigma-enriched signalling proteins that may function in compatible pollen responses, and this search led to the identification of the stigma-specific *BKN1* in *A. thaliana* Col-0. However, the *bkn1* mutants in the Col-0 ecotype did not display any detectable changes in compatible pollen responses and only a mild-hydration defect was observed when wild-type Col-0 pollen was placed on mutant *bkn2* stigmas (a tandemly linked paralogue). In addition, the level of impairment did not increase for the *bkn1-bkn2* double mutant stigmas. This was rather puzzling since *BKN2* was only expressed at a low level in the stigma, particularly in comparison to *BKN1*. Further investigations uncovered that the *BKN1* gene in most *A. thaliana* ecotypes carried two indels, ΔT128 and ^A597, that would cause frameshifts in the BKN1 coding region resulting a loss of full-length protein. Interestingly, three ecotypes, Hh-0, Dju-1 and Västervik, were found to carry two ORF-restoring SNPs (^T128 and ΔA597) and predicted to encode a full-length BKN1 protein. Furthermore, two outcrossing *Arabidopsis* species, *A. lyrata* and *A. halleri*, also carry *BKN1* orthologues that are predicted to encode full-length BKN1 proteins. These combined results raise a few questions for further investigation. Does the mild hydration phenotype associated with *bkn2* mutants point to a related function for *BKN1* that was lost during the transition to selfing for *A. thaliana*? For example, is BKN1’s stigma function related to pollen-stigma interactions in outcrossing species? The evolution from outcrossing to selfing occurs under different selective mechanisms, such as reduced access to pollinators or population bottlenecks associated with colonization of new environments, and is associated with the loss of self-incompatibility in Brassicaceae species (reviewed in [92, 93]). The transition to selfing is also associated with a change in several floral traits termed the ‘selfing syndrome’ and includes changes in flower size and shape (small flowers) and reduced pollen numbers, as seen in comparisons between outcrossing *A. lyrata/A. halleri* and selfing *A. thaliana* [93–95]. Other changes associated with the ‘selfing syndrome’ include loss of pollinator attraction traits (reviewed in [93, 95]).

Specifically related to selfing, there are traits, in the addition to the loss of self-incompatibility, that can be modified to improve self-pollination in the transition to selfing. These include dichogamy (temporal differences between time of pollen release and stigma receptivity), herkogamy (height differences between to stigma and anthers to prevent self-pollination) and anther orientation (anther surface undergoing dehiscence is oriented away from stigma) [93, 95, 96]. *A. lyrata* and *A. halleri* are self-incompatible species, whereby they exhibit a tight control of outcrossing through the linked *S-locus protein 11/S cysteine-rich* and *S receptor kinase* polymorphic genes (reviewed in [16]). With both species carrying intact *BKN1* genes, it would be of interest to investigate a potential role in stigma-pollen interactions in the context of these different traits designed to avoid self-pollination. As well, some North American *A. lyrata* populations around the Great Lakes region have also shifted towards self-compatibility, but show no significant changes towards the selfing syndrome [97–99]. These self-compatible *A. lyrata* may also be interesting to compare loss-of-function *BKN1* mutations in the context of self-pollination. Finally, the recent discovery of a role for BKN2/SZE2 in plant immunity [91] opens another direction of inquiry. Related to this, dual roles have been uncovered for other signaling proteins in both plant reproduction and pathogen responses [5]. The relative ease with which CRISPR/Cas9 technology can be used to create loss-of-function mutants opens the door to asking these questions regarding *BKN1* function in other *A. thaliana* ecotypes and *Arabidopsis* species in the future.

## Conclusions

In this study, we have identified a novel family of Brassicaceae-specific pseudokinase genes, termed *BRASSIKINs*, and specifically focused on the function of the tandemly linked *BKN1* and *BKN2* genes, in the context of pollen-stigma interactions in *A. thaliana* Col-0. CRISPR deletion mutants were generated, and very mild hydration defects were observed for wild-type Col-0 pollen when placed on the *bkn2* and *bkn1/2* mutant stigmas. Polymorphisms leading to premature stop codons were uncovered for *BKN1* in many *A. thaliana* ecotypes including Col-0 while absent in outcrossing *Arabidopsis* species. Thus, future studies should focus on examining *BKN1* function in other *A. thaliana* ecotypes and *Arabidopsis* species.

## Methods

### Plant materials and growth conditions

Seeds for the *A. thaliana bkn1–1* T-DNA insertion mutant (Col-0, SALKseq_039336), and the *A. thaliana* Hh-0 (CS76512), Västervik (CS78834), Dju-1 (CS78896) and Bela-1 (CS76696) ecotypes were obtained from Arabidopsis Biological Resource Center (ABRC). Seeds for the *A. thaliana* Col-0 ecotype and *N. benthamiana* were obtained from Dr. Nambara and Dr. Yoshioka, respectively (University of Toronto). *A. thaliana* seeds were sterilized and cold stratified for at least 2 days at 4 °C, then transferred to soil or plated on ½ Murashige and Skoog (MS) medium plates with 0.4% (w/v) phytoagar at pH 5.8 at 22 °C under 16 h light. After 7–10 days, seedlings were transferred to soil supplemented with 1 g/L 20–20-20 fertilizer and grown at 22 °C under 16 h light. For the *A. thaliana bkn1–1* T-DNA insertion mutant (SALKseq_039336), homozygous mutants were confirmed by PCR, and the location of the T-DNA was verified by sequencing of PCR products. *N. benthamiana* seeds were cold stratified for several days and planted directly on soil, and grown at 22 °C under 16 h light conditions. Humidity was monitored and maintained at between 20 to 60% relative humidity in the growth chambers.

### Plasmid construction and plant transformation

The 371 bp *BKN1* predicted promoter consists of the untranslated region immediately following the *BKN2* coding region to the *BKN1* start codon. The *BKN2* predicted promoter covers 465 bp upstream of the *BKN2* start codon, including the 3’UTR for At5g11412. The *BKN1* and *BKN2* 5′ predicted promoter regions were synthesized by GeneArt gene synthesis services (ThermoFisher Scientific). The promoters were cloned into the pORE-R2 vector upstream of the GUS coding region through *Xho*I and *Not*I sites, [100], transformed into *Arabidopsis thaliana* Col-0 by floral dip [101]. T1 seeds were selected for kanamycin resistance on ½ MS medium plates containing 50 μg/ml kanamycin. Inflorescence or stage 12 flowers from several T1 plants were stained for GUS activity (see below).

For the CRISPR/Cas9 generated mutants, a two-sgRNA (single guide RNA) system was used to generate genomic deletions in the *BKN1* and *BKN2* genes [48]. The CRISPR sgRNA sequences targeting *BKN1* or *BKN2* were selected using the CHOPCHOP software to search for sequences adjacent to PAM sites and avoid potential off-targets in the *A. thaliana* genome [102]. PCR fragments containing the two sgRNAs (See Additional file 4: Table S3 for primer sequences), along with the promoter and terminator sequences were generated from the pCBC DT1T2 vector template using Phusion polymerase (ThermoFisher Scientific). The purified fragments containing the two sgRNAs were cloned into the final vector pBEE401E using a golden gate reaction with BsaI enzyme. This vector was modified to carry the Basta resistance marker (*BlpR* from pBUE411 [103]) rather than the original *HygR* marker in pHEE401E [36, 48]. Constructs were transformed into *Agrobacteria* by electroporation, which were then used to transform *A. thaliana* Col-0 by floral dip [101]. T1 seeds were cold stratified and sown on soil as previously described [26]. Once seedlings had germinated, selection for Basta™ herbicide resistance was carried out, and resistant seedlings were transplanted, and PCR screened for the Basta™ selection marker and for genomic deletion. T1 plants were analyzed with primers pairs designed to amplify inside or outside of the deletion regions for *BKN1/BKN2* to identify heterozygous mutants, and homozygous mutants carrying the respective gene deletions were identified in subsequent generations (T2-T5) for phenotyping. PCR products covering the deletions were sequenced to confirm the locations of each independent deletion mutation. For both *BKNs*, two constructs carrying different sgRNA target sites were screened for deletions in the T1 (Additional file 4: Table S3). The *BKN1_CR #1* and *BKN1_CR #3* constructs produced the *bkn1–2* and *bkn1–3* mutants, respectively. The *BKN2_CR #3* construct produced the *bkn2–1* and *bkn2–2* mutants. To generate double mutants, transgene free *bkn1–3* mutants were transformed with the *BKN2_CR #2* construct to produce three new *bkn2* mutants, *bkn2–3*, *bkn2–4* and *bkn2–5*, in the *bkn1–3* background.

To clone cDNAs for *A. thaliana BKN2* (Col-0, At5g11410), *A. lyrata BKN1* (AL6G22040.t1) and *A. lyrata BKN2* (AL6G22050.t1), RT-PCR was conducted on RNA extracted from top ½ pistil tissue. The *A. thaliana* Hh-0 *BKN1* cDNA was cloned from stage 12 flower bud RNA. The *BKN* clones were introduced into the TOPO entry clone using the PCR8/GW TOPO cloning kit (ThermoFisher Scientific). To generate the Al-BKN2(G2A), Al-BKN2(C4A), and Al-BKN2(G2A, C4A) constructs, the myristoylation (G2) and palmitoylation (C4) sites at the N-terminus of *A. lyrata* BKN2 were disrupted by PCR with primers to replace the G2 and C4 sites (Additional file 4: Table S3). Gateway reactions were carried out using LR clonase II enzyme (ThermoFisher) into the destination vector pEARLEYGATE 101 containing a C-terminal YFP (Earley et al., 2006). Plasmids were then transformed into *Agrobacterium* GV2260 by electroporation for the agroinfiltration experiments. Leaves 3 or 4 from 5 week-old *N. benthamiana* leaves were transformed by agroinfiltration as described in the protocol by Sparkes et al. [104].

### Promoter-GUS staining

Inflorescences or stage 12 flowers from the *BKN1* and *BKN2* promoter-GUS transgenic plants were incubated in GUS solution overnight at 37 °C according to the protocol used in Wang et al. [15]. Tissues were then fixed in ethanol:glacial acetic acid and cleared with chloral hydrate solution as described by [105]. Tissues were mounted in 30% glycerol and images were taken on a Nikon sMz800 microscope.

### Confocal microscopy

At 24 to 48 h post-infiltration, leaf disks were cut from *N. benthamiana* and visualized using a Leica TCS SP8 confocal microscope. Image processing was done using the Leica LAS AF lite software. Plasmolysis was achieved by treatment with 0.8 M mannitol as described by Lang et al. [106]

### Expression profiling, multiple sequence alignments and phylogenetic analyses

The BAR Expression Angler tool [39] (http://bar.utoronto.ca/) was used to search for stigma-enriched signalling proteins as previously described [36]. Briefly, the stigma-specific SLR1 gene as the bait (At3g12000, [40]) to search the AtGenExpress Plus-Extended Tissue Compendium dataset [39, 41]. Expression profiling of the BKN genes for Additional file 1: Figure S1 came from three additional transcriptome datasets: the TRAVA RNA-Seq dataset (http://travadb.org/ [44]), the stigmatic papillae RNA-Seq dataset [43] and the stigma microarray datasets [35]; and the data was displayed using the HeatMapper Plus tool [39].

For ecotype polymorphism searches of the 1135 genomes [50], two different databases were used to retrieve the BKN1 genomic sequences: *1001 Genomes* (https://1001genomes.org/) and *Salk Arabidopsis 1001 Genomes* (http://signal.salk.edu/atg1001/index.php). Hh-0 was the first ecotype identified to carry the T128 and ΔA597 to encode a full-length BKN1 protein. The MEGA7 software [63] was used to produce multiple protein sequence alignments of the BKN1 genomic sequences retrieved from the 1135 ecotype genomes. The Col-0 and Hh-0 BKN1 cDNA sequences were included to remove introns and locate the position of the two SNPs (Additional file 5 and Additional file 6). The two SNP regions were copied from the alignment into an excel file for further analysis (see Additional file 3: Table S2). Genomic DNA samples were used to PCR amplify and sequence the BKN1 gene for the Västervik, Dju-1 and Bela-1 ecotypes (Additional file 1: Figure S5).

For the phylogenetic analysis of Brassicaceae BKNs (Fig. 6), amino acid sequences (Additional file 7) were obtained from TAIR (*A. thaliana*) [49]; Phytozome (*A. lyrata, B. oleracea capitata, B. stricta, E. salsugineum -* formerly *T. halophila, C. rubella, C. grandiflora, C. papaya*) [107]; NCBI (*A. alpina, B. cretica, B. oleracea* cv TO1000, *T. hassleriana*) [108]; EnsemblPlants (*A. halleri, B. oleracea* cv TO1000) [109]; BRAD (*A. arabicum*) [110]; or thellungiella.org (*S. parvula* - formerly *T. parvula*) servers using blastp or tblastn searches for genes similar to the BKNs or other RLCKs. For the phylogenetic analysis of Arabidopsis RLCK VII members (Additional file 1: Figure S8), the RLCK VII members defined by Lehti-Shiu and Shiu [51] were used, and amino acid sequences (Additional file 8) were retrieved from TAIR [49]. The MEGA7 software [63] was used to produce multiple protein sequence alignments using ClustalW [62]. The ClustalW alignments were trimmed at the N-and C-terminus and then used to generate a consensus tree by the Maximum Likelihood method [64] with 1000 bootstrap replicates [65] in MEGA7. Alignments in Additional file 1: Figure S3, S4, S5 and S6 were generated in MEGA7 and formatted with the *Multiple Align Show* tool (http://www.bioinformatics.org/sms/ [111]), using groupings of amino acids based on their side chains [112]. See additional files for all amino acid sequences and alignments.

### RT-PCR and quantitative RT-PCR software

Anthers and pistil tissues (top-half: stigmas, bottom-half: ovaries) and were collected from stage 12 flower buds; leaves and roots were collected from 2-week-old *A. thaliana* seedlings for RT-PCR and quantitative RT-PCR applications. RNA was extracted using a modified protocol of the SV total RNA extraction kit (Promega) which included vigorous grinding of plant tissue in liquid nitrogen. Next, cDNA synthesis was carried out using Superscript III reverse transcriptase (ThermoFisher) and oligo dT primers. The cDNA was then used in RT-PCR reactions with Taq polymerase, and quantitative RT-PCR reactions with PowerUp 2x SYBR Green master mix (ThermoFisher) (primers listed in Additional file 4: Table S3).

### Assays for pollen hydration, pollen adhesion and pollen tube growth, and seed set

Stage 12 flower buds were emasculated and carefully wrapped with plastic wrap and allowed to mature overnight. For pollen hydration, the next day, pistils were mounted upright in ½ MS medium and hand-pollinated with a small amount of Col-0 pollen. Pictures were taken immediately at 0 min and again at 10 min post-pollination using a Nikon sMz800 microscope at 6x magnification with a 1.5x objective. Pollen grain diameter was measured laterally using the Nikon digital imaging software for 10 random pollen grains per pistil, 3 pistils per genotype. All pollinations were performed under an ambient humidity lower than 60% to avoid spontaneous water uptake from the surrounding environment.

For pollen adhesion and pollen tube growth, the next day, pistils were carefully unwrapped and lightly pollinated with Col-0 pollen, or transgenic pollen for reciprocal crosses. At 2 h post-pollination, pistils were collected, fixed and stained with aniline blue to stain the callose deposited by pollen tubes, as described by Safavian et al. (2015). Pollinated pistils were imaged using a Zeiss Axioskop2Plus microscope under brightfield to count the number of pollen grains adhered, and under UV fluorescence to assess pollen tube growth. Pollen adhesion was quantified for *n* = 10 pistils for each cross.

For seed set, late stage 12 buds were emasculated and hand-pollinated with Col-0 pollen for Col-0 pistils and for each transgenic line. Hand pollinations were marked with thread and siliques were allowed to mature fully over several days. Prior to senescence, green siliques were removed, and sliced longitudinally to count the number of developing seeds. 10 siliques were counted for each pollination.

### In vitro BKN2 Palmitoylation assay

At-BKN2 and Al-BKN2 gateway entry clones were recombined into the Gateway destination vector pYES-DEST52 (C-terminal V5 tagged, Invitrogen) to create yeast expression vectors pYES-Al-BKN2 and pYes-At-BKN2 respectively. Wild-type yeast BY4741 (MATa his3Δ1 leu2Δ0 met15Δ0 ura3Δ0) cells were transformed and grown at 25 °C in selective minimal media minus uracil to select transgenic yeast cells. To induce protein expression, the transformed yeast cells were grown in minimal liquid media containing 2% galactose. The palmitoylation assay was carried out by the Acyl-RAC method [82, 113]. Briefly, total proteins were lysed and recovered by acetone precipitation. Free –SH was blocked with 1% methyl methanethiosulfonate (MMTS), and samples were then treated with 1 M hydroxylamine, pH 7.5 (+ NH_2_OH) to remove palmitoylate and to expose free thiols at the palmitoylation sites. In the negative control (−NH_2_OH), 1 M Tris (pH 7.5) was added. Palmitoylated proteins were captured on thiopropyl sepharose beads (Sigma), and the presence of BKN2 proteins were detected by ECL western blotting with anti-V5 primary and HRP-conjugated secondary antibodies (CWBio, China).

## Supplementary information

**Additional file 1: Figure S1.** Tissue expression profiles of the BKNs in different transcriptome datasets. **Figure S2** Analysis of the *bkn1–1* T-DNA mutant. **Figure S3.** Sequence Alignment of *Arabidopsis BKN1* coding sequences. **Figure S4.** *Arabidopsis* genomic regions for the *BKN* genes and amino acid alignment for the *BKN1* annotations. **Figure S5.** *A. thaliana*, *A. lyrata* and *A. halleri* amino acid alignments for BKN1, BKN2 and BKN3. **Figure S6.** Alignment of BKN1 coding sequences from four *A. thaliana* ecotypes. **Figure S7.** Pollen Hydration Assays in the Col-0 and Hh-0 ecotypes at 10 min post-pollination. **Figure S8**. Phylogenetic analysis of the *Arabidopsis* RLCK subfamily VII members. **Figure S9** Amino acid sequence alignment of BRASSIKIN (BKN) sequences with CASTAWAY (CST) sequences. **Figure S10.** Confocal microscopy imaging of *N. benthamiana* leaves infiltrated with C-terminal BKN:YFP fusion protein constructs and plasmolysed with 0.8 M mannitol

**Additional file 2: Table S1.** BAR Expression Angler using the stigma specific *SLR1* gene as a bait

**Additional file 3: Table S2.** Polymorphism searches across the 1001 genomes for At-*BKN1*

**Additional file 4 Table S3.** Primers Used

**Additional file 5.** RLCK Amino acid sequences for Fig. 6 phylogenetic tree

**Additional file 6.** RLCK Amino acid sequences for Additional; file 1: Fig. S7 phylogenetic tree

**Additional file 7.** RLCK Amino acid sequences for Fig. 6 phylogenetic tree

**Additional file 8.** RLCK Amino acid sequences for Additional file 1: Fig. S7 phylogenetic tree

## Data Availability

All data is included in this manuscript. Constructs and seeds are available upon request from the corresponding author.

## Abbreviations

BIK1: BOTRYTIS-INDUCED KINASE1
BKN: BRASSIKIN
CST: CASTAWAY
MMTS: Methyl methanethiosulfonate
MS: Murashige and Skoog
PBL: PBS1-Like
PCP-B: Pollen Coat Protein-B
RLCK: Receptor-Like Cytoplasmic Kinase
SPIK: SHAKER POLLEN INWARD K+ channel
