# Supplementary material for: Investigations into a putative role for the novel BRASSIKIN pseudokinases in compatible pollen-stigma interactions in Arabidopsis thaliana
Source: BMC Plant Biol. 2019 Dec 11;19:549. doi: 10.1186/s12870-019-2160-9 (PMC6907349; doi:10.1186/s12870-019-2160-9)
Supplement: Supplementary file 1 — Additional file 1: Figure S1. Tissue expression profiles of the BKNs in different transcriptome datasets. Figure S2 Analysis of the bkn1–1 T-DNA mutant. Figure S3. Sequence Alignment of Arabidopsis BKN1 coding sequences. Figure S4. Arabidopsis genomic regions for the BKN genes and amino acid alignment for the BKN1 annotations. Figure S5. A. thaliana, A. lyrata and A. halleri amino acid alignments for BKN1, BKN2 and BKN3. Figure S6. Alignment of BKN1 coding sequences from four A. thaliana ecotypes. Figure S7. Pollen Hydration Assays in the Col-0 and Hh-0 ecotypes at 10 min post-pollination. Figure S8. Phylogenetic analysis of the Arabidopsis RLCK subfamily VII members. Figure S9 Amino acid sequence alignment of BRASSIKIN (BKN) sequences with CASTAWAY (CST) sequences. Figure S10. Confocal microscopy imaging of N. benthamiana leaves infiltrated with C-terminal BKN:YFP fusion protein constructs and plasmolysed with 0.8 M mannitol [file 12870_2019_2160_MOESM1_ESM.pdf]

Fig. S1 Tissue expression profiles of the *BKNs* in different transcriptome datasets.

TRAVA - transcriptome variation analysis (RNA-Seq)
(Klepikova et al., 2016) http://travadb.org/
Relative read counts. Read counts normalized by TMM. Format: average value.

	BKN1	*BKN2*	*BKN3*
**Tissue**	At5g11400	At5g11410	At5g11360
Stigmatic tissue - 6th & 7th flowers	4977	220	156
Ovules - 6th & 7th flowers	7	4	410
Carpels - 6th & 7th flowers (ovules/stigmas removed)	9	6	131
Opened anthers, with pollen	1	1	20
Anthers of the mature flower (before opening).	0	0	9
Carpels of the mature flower (before pollination	196	13	249
Stamen filaments of the mature flower	0	4	34
Petals of the mature flower	0	0	10
Sepals of the mature flower	0.5	3	6
Anthers of the young flower	0.9	1	10
Carpels of the young flower	15	14	278
Sepals of the young flower	0	12	10
Flower 1	30	21	317
Flower 2	96	13	165
Flower 3	150	14	148
Flower 4	109	21	197
Flower 5	87	19	222
Flower 6-8	29	16	171
Flower 9-11	13	24	166
Flower 12-14	4	17	63
Flower 15-18	0.7	13	34
Flower 19+	0.3	13	44
Axis of the inflorescence	0.4	32	1
Inflorescence meristem at 13 days after germination	3	53	23
Inflorescence meristem at 14 days after germination	0.8	37	22
Inflorescence meristem at 15 days after germination	0.4	23	16
Inflorescence meristem at 16 days after germination	0	13	26
Pedicel	2	12	5
Internode	0	81	2
Senescent internode.	0	98	5
Leaf blade, intermediate 1	0	13	6
Leaf blade, intermediate 2	0	10	3
Leaf blade of the mature leaf	0.5	6	5
Leaf blade of the young leaf	0	4	9
Whole mature leaf	0.4	24	5
Petiole, intermediate 1	0	27	3
Petiole, intermediate 2	0.5	79	3
Petiole of the mature leaf	0	145	4
Petiole of the senescent leaf	0.5	84	3
Petiole of the young leaf	0	21	7
Leaf vein, intermediate 2	0	13	4
Vein of the mature leaf	0.9	56	4
Vein of the senescent leaf	1	33	5
SAM at 7 days after germination	0.4	197	4
SAM at 8 days after germination	6	51	11
SAM at 9 days after germination	3	48	7
Meristem at 10 days after germination	5	201	56
Meristem at 11 days after germination	5	122	27
Meristem at 12 days after germination	1	50	23
Root without apex	0	2	7
Root apex	0	0	3
Seedling cotyledons	0	14	8
Seedling hypocotyl	0	0	7
Seedling meristem	0	0	13
Seedling root	0	0	2
Young seeds 1	6	6	1334
Young seeds 2	3	8	1430
Young seeds 3	3	6	1259
Young seeds 4	3	7	888
Young seeds 5	2	3	677
Seeds 1	0	1	204
Seeds 3	2	2	794
Seeds 5	4	4	1215
Seeds 7	3	9	1481
Dry seeds	2	0	8
Germinating seeds 1 (first day after soaking)	0	10	16
Germinating seeds 2 (second day after soaking)	0	0.5	1
Germinating seeds 3 (third day after soaking)	0	46	11
Silique 2	1	13	187
Silique 4	0	14	312
Silique 6	0.9	8	546
Silique 8	1	11	445
Pod of the silique 1	0	53	45
Pod of the silique 3	0	13	84
Pod of the silique 5	1	10	145
Pod of the silique 7	0	13	170
Senescent silique 2	0	1	32
Pod of the senescent silique 1.	0	31	196
Seeds of first yellowing silique.	0	1	14

Klepikova et al: A high resolution map of the Arabidopsis thaliana developmental transcriptome based on RNA-seq profiling. Plant J 2016, 88(6):1058-1070.

Scale
>2000
1800
1600
1400
1200
1000
800
600
400
200
0

Pollinated Stigmas Datasets (microarrays) (Iwano et al., 2014)

		BKN1	BKN2	*BKN3*
Tissue samples	Treated with	At5g11400	At5g11410	At5g11360
Stigmas, 0 min	Pollen grains	318	68	50
Stigmas, 15 min	Pollen grains	251	57	67
Stigmas, 0 min	Pollen Coating	581	72	57
Stigmas, 15 min	Pollen Coating	285	62	53

Iwano et al: A Pollen Coat-Inducible Autoinhibited Ca2+-ATPase Expressed in Stigmatic Papilla Cells Is Required for Compatible Pollination in the Brassicaceae. Plant Cell 2014, 26(2):636-649.

Scale
>500
450
400
350
300
250
200
150
100
50
0

Stigmatic papillae RNA-Seq (Gao *et al.* , 2018)

Average of 3 replicates for each stage

	BKN1	BKN2	*BKN3*
	At5g11400	At5g11410	At5g11360
Stage 1	207	18	2
Stage 2	172	16	4
Stage 3	52	6	5

Stage 1 = 1 day after emasculation
(Stage 13, stigmatic papillae are receptive)
Stage 2 = 2 days after emasculation
Stage 3 = 3 days after emasculation (onset of papillar cell death)

Gao et al: KIRA1 and ORESARA1 terminate flower receptivity by promoting cell death in the stigma of Arabidopsis. Nat Plants 2018, 4(6):365-375.

Scale
>250
225
200
175
150
125
100
75
50
25
0

Heatmaps generated with BAR HeatMapper Plus Tool http://bar.utoronto.ca/

Toufighi et al: The Botany Array Resource: e-Northerns, Expression Angling, and Promoter analyses. Plant Journal 2005, 43(1):153-163.

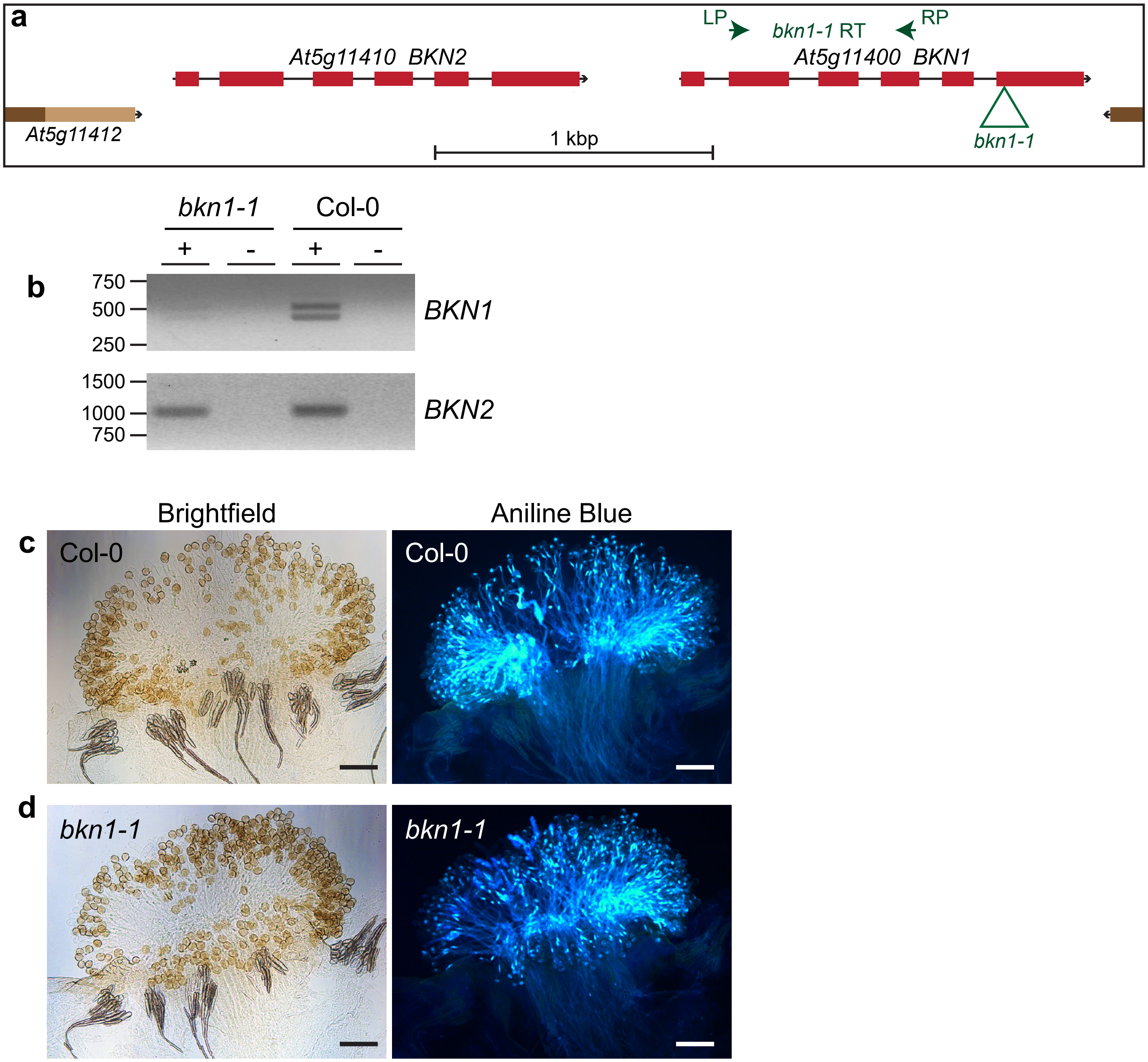

Fig. S2 Analysis of the *bkn1-1* T-DNA mutant

a. Gene structures for the tandemly-linked BKN1 and BKN2 genes. The T-DNA insertion in the bkn1-1 mutant (SALKseq_039336) is located in the sixth exon. Primers used for expression analysis of bkn1-1 (LP/RP, in green).

b. RT-PCR analysis for the *bkn1-1* T-DNA mutant and Col-0 using primers upstream of T-DNA insertion shows a reduction of *BKN1* expression in *bkn1-1* not for Col-0.

c-d. Aniline blue-stained pistils, following 2 hours pollination with Col-0 pollen. Brightfield images (left) show pollen grains adhered to the stigmatic papillae, and fluorescent images (right) show aniline blue stains of callose deposits in the pollen tubes. Representative images of pollinated Col-0 pistil (a) and *bkn1-1* T-DNA line (b) show wild-type pollen adhesion and pollen tube penetration. Scale bar = 100μm.

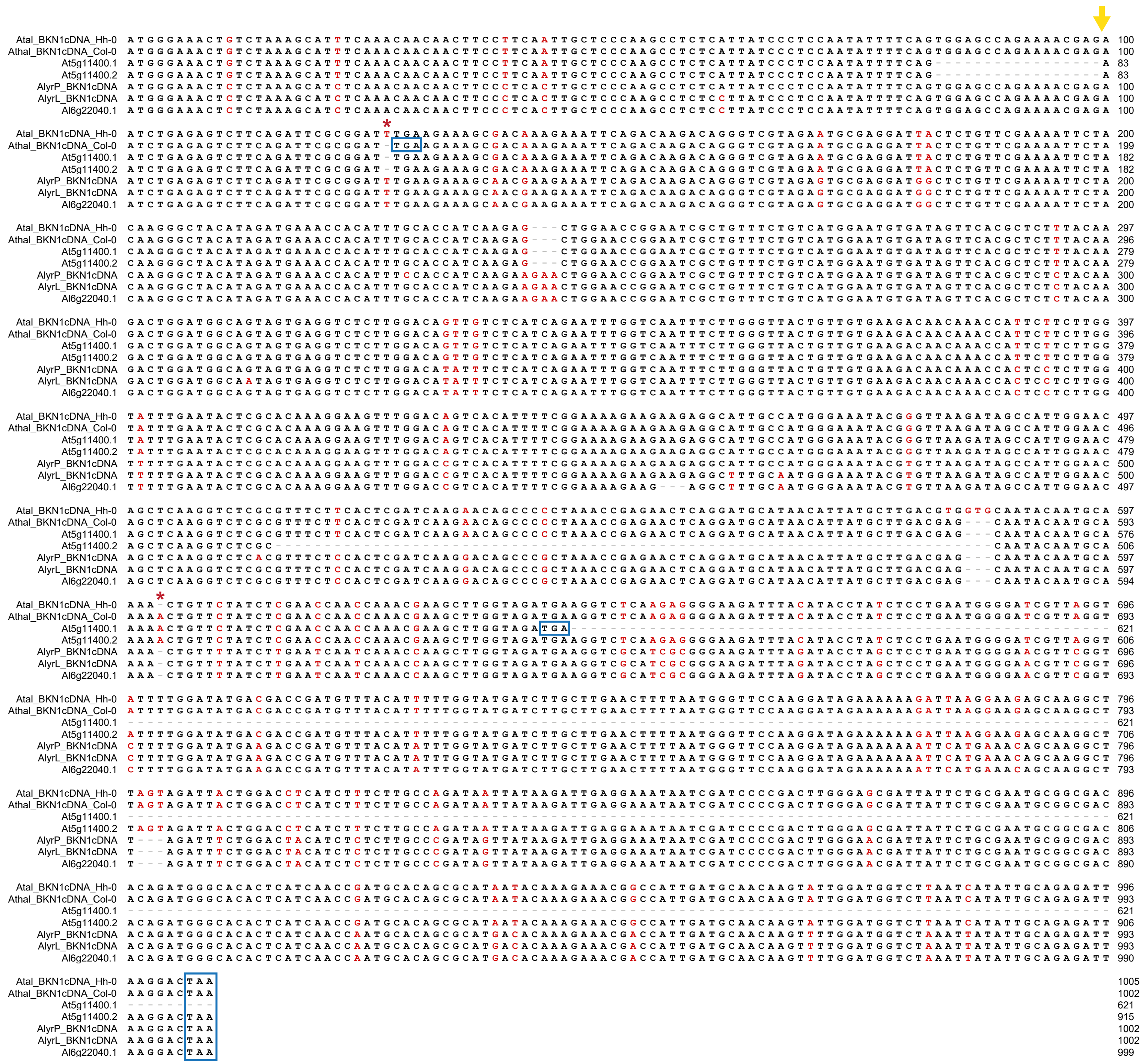

Fig. S3 Sequence Alignment of Arabidopsis *BKN1* coding sequences.
BKN1 cDNAs were cloned from two different *A. thaliana* ecotypes, Col-0 and Hh-0 sequences and from two different *A. lyrata* sub-species, *A. lyrata lyrata* and *A. lyrata petrea*. The cDNA sequences are aligned to the predicted *BKN1* annotations from TAIR/Araport, At5g11400.1 and At5g11400.2, and from Phytozome, Al6g22040.1. Stop codons are boxed. Red astericks (*) mark two indels in Col-0 BKN1 (ΔT128, ^A597) when compared to Hh-0 BKN1 and Al-BKN1, that results in a premature stop codon (cDNA sequence). Based on the reduced carpel RNA-Seq coverage and splice junctions mapped to the 5' end of the BKN1 exon 2 (Fig. 2), there may be alternate splice sites in use (yellow arrow). There are several potential splice sites in this region that will restore the At-BKN1 reading frame to produce a longer protein as predicted for the At-BKN1.1 and At-BKN1.2 annotations.

a

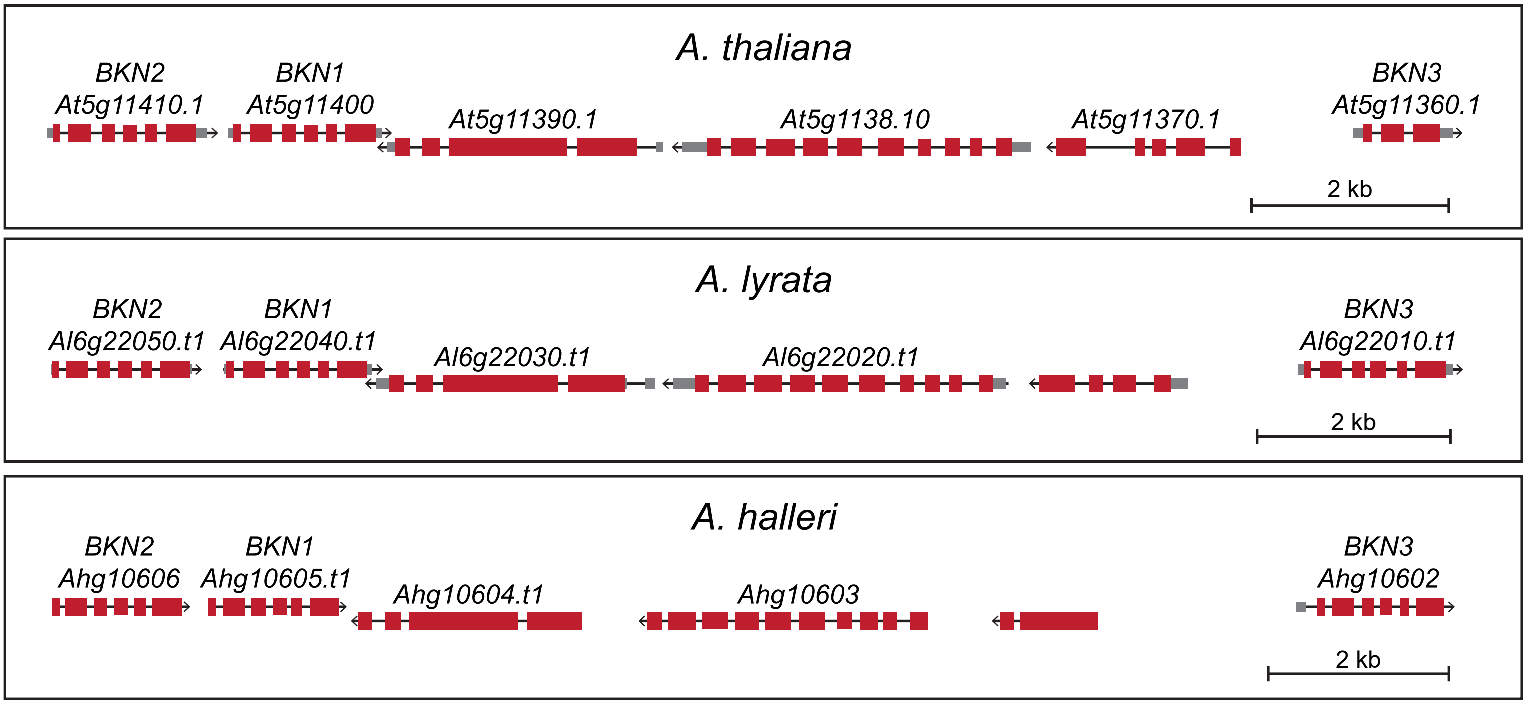

b

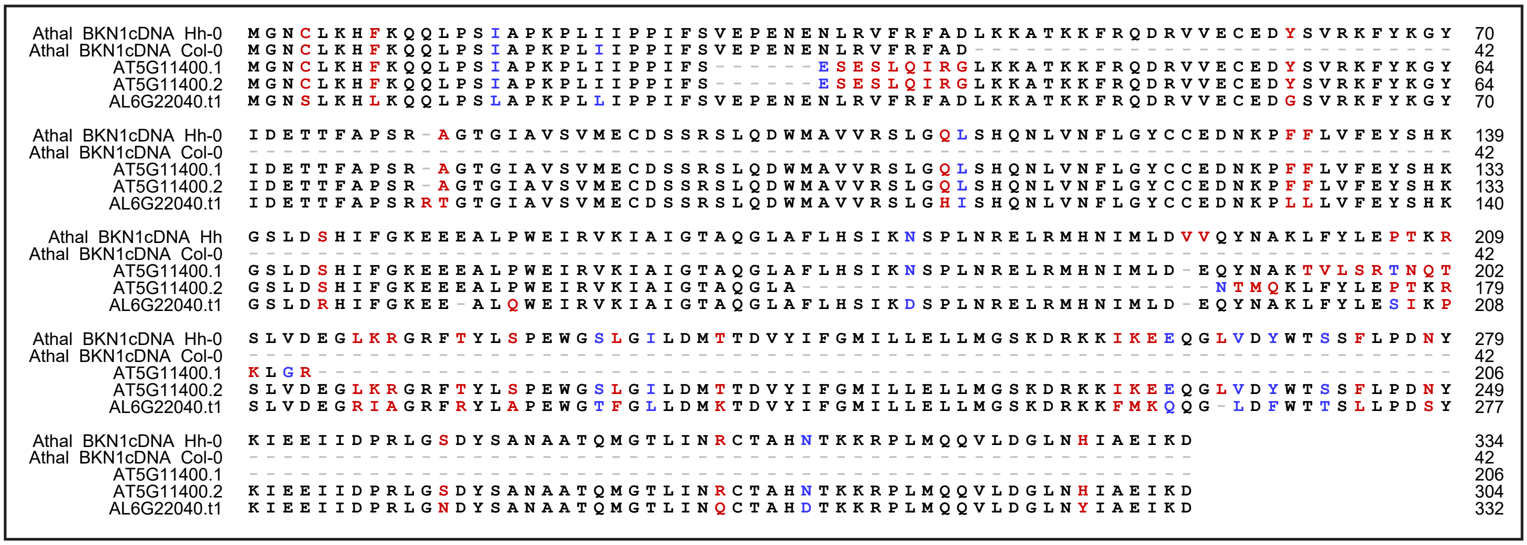

Fig. S4 *Arabidopsis* genomic regions for the *BKN* genes, and amino acid alignment for the BKN1 annotations. **a.** *A. thaliana*, *A. lyrata* and *A. halleri* genomics regions convering the *BKN1*, *BKN2* and *BKN3* genes were drawn to scale using data from Araport [49], Phytozome [102], EnsemblPlants [104] and NCBI [103]. **b.** Amino acid sequences for the predicted *A. thaliana* and *A. lyrata BKN1* annotations were aligned using ClustalW in MEGA7.0 [96], and the alignments were formatted using Multiple Align Show [100]. Identical amino acids are shown in black text, similar amino acids are shown in blue text, and no identity are shown in red text. Percentage of sequences that must agree for identity or similarity coloring was set at 60%. Similar amino acids were grouped as: GAVLI (aliphatic), FYW (aromatic), CM (sulfur-containing), ST (hydroxyl), KRH (basic), DENQ (acidic and their amide), and P (cyclic) [110].

a

```
Athal_BKN1cDNA_Hh   MGNCLKHFKQQLPSIAPKPLIIPPIFSVEPENENLRVFRFADLKKATKKFRQDRVVECEDYSVRKFYKGY   70
AlyrP_BKN1cDNA      MGNSLKHLKQQLPSLAPKPLIIPPIFSVEPENENLRVFRFADLKKATKKFRQDRVVECEDGSVRKFYKGY   70
AlyrL_BKN1cDNA      MGNSLKHLKQQLPSLAPKPLLIPPIFSVEPENENLRVFRFADLKKATKKFRQDRVVECEDGSVRKFYKGY   70
AL6G22040.t1        MGNSLKHLKQQLPSLAPKPLLIPPIFSVEPENENLRVFRFADLKKATKKFRQDRVVECEDGSVRKFYKGY   70
Ahg10605.t1         MGNSLKHLKQQLPLLAPKPLITPPIFSVEPENENLRVFRFADLKKATKKFRQDRVVECEDGSVRKFHKGY   70

Athal_BKN1cDNA_Hh   IDETTFAPSR-AGTGIAVSVMECDSSRSLQDWMAVVRSLGQLSHQNLVNFLGYCCEDNKPFFLVFEYSHK  139
AlyrP_BKN1cDNA      IDETTFPPSRRTGTGIAVSVMECDSSRSLQDWMAVVRSLGHISHQNLVNFLGYCCEDNKPLLLVFEYSHK  140
AlyrL_BKN1cDNA      IDETTFAPSRRTGTGIAVSVMECDSSRSLQDWMAIVRSLGHISHQNLVNFLGYCCEDNKPLLLVFEYSHK  140
AL6G22040.t1        IDETTFAPSRRTGTGIAVSVMECDSSRSLQDWMAVVRSLGHISHQNLVNFLGYCCEDNKPLLLVFEYSHK  140
Ahg10605.t1         IDETTFAPSRRTGAGIAVSVMECDSSRSLQDWMAVVRSLGQMSHQNLVDFLGYCYEDNKPLFLVFEYSHK  140

Athal_BKN1cDNA_Hh   GSLDSHIFGKEEE-ALPWEIRVKIAIGTAQGLAFLHSIKNSPLNRELRMHNIMLDVVQYNAKLFYLEPTK  208
AlyrP_BKN1cDNA      GSLDRHIFGKEEE-ALPWEIRVKIAIGTAQGLTFLHSIKDSPLNRELRMHNIMLD-EQYNAKLFYLESIK  208
AlyrL_BKN1cDNA      GSLDRHIFGKEEE-ALQWEIRVKIAIGTAQGLAFLHSIKDSPLNRELRMHNIMLD-EQYNAKLFYLESIK  208
AL6G22040.t1        GSLDRHIFGKEE--ALQWEIRVKIAIGTAQGLAFLHSIKDSPLNRELRMHNIMLD-EQYNAKLFYLESIK  207
Ahg10605.t1         GSLDSHIFGKEEEEALPWEIRVKIAIGTAQGLAFLHSIKTRPLHRELRMHNIMLD-EQYNAKLFYLESIK  209

Athal_BKN1cDNA_Hh   RSLVDEGLKRGRFTYLSPEWGSLGILDMTTDVYIFGMILLELLMGSKDRKKIKEEQGLVDYWTSSFLPDN  278
AlyrP_BKN1cDNA      PSLVDEGRIAGRFRYLAPEWGTFGLLDMKTDVYIFGMILLELLMGSKDRKKFMKQQG-LDFWTTSLLPDS  277
AlyrL_BKN1cDNA      PSLVDEGRIAGRFRYLAPEWGTFGLLDMKTDVYIFGMILLELLMGSKDRKKFMKQQG-LDFWTTSLLPDS  277
AL6G22040.t1        PSLVDEGRIAGRFRYLAPEWGTFGLLDMKTDVYIFGMILLELLMGSKDRKKFMKQQG-LDFWTTSLLPDS  276
Ahg10605.t1         PSLVDEGLIAGRFRYLAPEYGTFGLLDMKTDVYVFGMILLELLMGSKDRKKIKNKEG-LDFWTTSLLPDD  278

Athal_BKN1cDNA_Hh   YKIEEIIDPRLGSDYSANAATQMGTLINRCTAHNTKKRPLMQQVLDGLNHIAEIKD                334
AlyrP_BKN1cDNA      YKIEEIIDPRLGNDYSANAATQMGTLINQCTAHDTKKRPLMQQVLDGLNYIAEIKD                333
AlyrL_BKN1cDNA      YKIEEIIDPRLGNDYSANAATQMGTLINQCTAHDTKKRPLMQQVLDGLNYIAEIKD                333
AL6G22040.t1        YKIEEIIDPRLGNDYSANAATQMGTLINQCTAHDTKKRPLMQQVLDGLNYIAEIKD                332
Ahg10605.t1         YKIEEIIDPRLGNDYSANAATQMGTLINRCTAHDTKKRPLMQQVLDGLNYIAEIKD                334
```

b

```
Athal_BKN2cDNA_Col-0  MGNCLKPLKEQPPSASPKPLTIPSSSVEPVKENLKEFRFAELNKATKRFRKYMVIKGNDNGFTRTFYEGC   70
AlyrP_BKN2cDNA        MGNCLKPPK--PSSASPKPLINPPISGESENEYLREFNFAKLSKATKKFRQYMIIKGNDNGCTRTFYQGY   68
AL6G22050.t1          MGNCLKPLK--PSSASPKPLINPPISGESKNEYLREFNFAKLSKATKKFRQYMIIEGNDNGCTRTFYQGY   68
Ahg10606              MGNCLKPLK--PSPASPKPLINPPIS---ENEYLREFSFAKLSKATKKFRQYMVIKGNDNGCTRTFYQGY   65

Athal_BKN2cDNA_Col-0  INETTFAPSRTGITVSVMECYQDNSQTLQDWKEEVKSLGRISHPNLVKLLGYCCEENKSFLVFEYLHKGS  140
AlyrP_BKN2cDNA        INETTFAPSRTGIAVSVLECYQDNSQTLQDWKEEVKSLGRISHPNLVKLLGYCCEENKSFLVFEYLHKGS  138
AL6G22050.t1          INETTFAPSRTGIAVSVLECYQDNSQTLQDWKEEVKSLGRISHPNLVKLLGYCCEENKSFLVFEYLHKGS  138
Ahg10606              INETTFAPSRTGIAVSVLECYQDNSQTLQDWKEEVKSLGRISHPNLVKLLGYCCEENKSYLVFEYLHKGS  135

Athal_BKN2cDNA_Col-0  LNRYIFGKEEEALPWETRVKIAIGAAQSIAFLHWVKNSALYRELRMYNILLDEHYNTKLFYLGSKKLCLL  210
AlyrP_BKN2cDNA        LDRHIFGKEEEALPWEIRVKIAIGAAQSIAFLHSVKNSALYRELRMYNILLDENYNTKLFYLGSKKLCLL  208
AL6G22050.t1          LDRHIFGKEEEALPWEIRVKIAIGAAQSIAYLHSVKNSALYRELRMYNILLDENYNTKLFYLGSKKLCLL  208
Ahg10606              LDRHIFGKEEEALPWETRVKIAIGAAQSIAFLHSVKNSALYRELRMFNILLDENYNTKLFYLGSKKLCLL  205

Athal_BKN2cDNA_Col-0  EESVTTAFIGRTVYIPPEYVISGHLGTKSDVYTFGVILLEILTGLKASDGKKNENMQSLHVWT-KPFLSD  279
AlyrP_BKN2cDNA        EESVTTAFIGRTVYLAPEYAISGHLGTKSDVYTFGVILFEILTGLKASDG--NKNMQSLHVWTTKPFLSD  276
AL6G22050.t1          EESVTTAFIGRTVYVPPEYIISGHLGTKSDVYTFGVILFEILTGLKASDGKKNENMQSLHVWT-KPFLSD  277
Ahg10606              EESVTTAFIGRTVYLAPEYAISGHLGTKSDVYTFGVILFEILTGLKASDGKKNENMQSLHIWT-KPFLSD  274

Athal_BKN2cDNA_Col-0  QSKIREIIDPRLGNDYPVNAATQMGKLIKRCIKLDTRKRPSMQQVFDGLNDIAEIKD               336
AlyrP_BKN2cDNA        QSKIREIIDPRLGNDYPVNAATQMGKLIKRCIKLDTRKRPSMQQVFDGLNDIAEIKD               333
AL6G22050.t1          QSKIREIIDPRLGNDYPVNAATQMGKLIKRCIKLDTRKRPSMQQVFDGLNDIAEIKD               334
Ahg10606              QSKIREIIDPRLGNDYPVNAATQMGKLIKRCIKLDTRKRPSMQQVLDGLNDIAEIKD               331
```

c

```
Athal_BKN3cDNA_Col-0  MGNIVKPFKQQPSSFAYQPLTVPLIPEVEAQSENLRVFSFKEWMKATKKSRQDRVVIRDDHYIR--SFYK   68
AL6G22010.t1          MGNIVKPFKQQPSSFAYQPLTVPLISEVEAQNENLRVFSFKEWMKATKKYRQDRVELCDNPYIRFRTFYK   70
Ahg10602              MGNIVKPFKQQPSSFAYQPLTVPLISEVGAQNENLRVFSFKEWMKATKKYRQDRVEICDKRYIRFRIFYK   70

Athal_BKN3cDNA_Col-0  GYIDNTTFAPSRTKTGTPVSVVEYLHGSSQALQEWVD---------------------------------  105
AL6G22010.t1          GYIDNTTFAPSRTKTGTPVSVVEYLHKSSQALQEWVEEVKSLERFSHPNLVKILGYCCEDNKSLLIILLY  140
Ahg10602              GYIDNTTFAPSRTKTGTPVSVVEYLHDSSQALQEWVEEVKSLERFSHPNLVKILGYCCEDNKSLLVLEDL  140

Athal_BKN3cDNA_Col-0  ----------------------------------------------------------------------  105
AL6G22010.t1          IE--FHVSLSTEEEGLPWAIRVKIAIGTAQGLAFFHSIMNTPLHLELRLHNIMLNEQYNAKLFYRESNKR  208
Ahg10602              HQGSLDHHIFGKEEALPWEIRVKIAIGTAQGLAFFHSIMNTPLHLELRLHNIMLNEQYNAKLFYLESNKR  210

Athal_BKN3cDNA_Col-0  ----------------------------------IFGVILLELLVGSQDRSTALKNQSS-DWTGSFLPDN  140
AL6G22010.t1          NCLEEGTFVALLKYMPPEPECAMSARLGIEADVYIFGVILLEILAGSKARSRNMKNQSSYDWTGSFLPDN  278
Ahg10602              NCLEEGAFVALIRYMP--PECAMSARLGMEADVYIFGVILLEILAGSKARSRNMKNQSSDDWTGSFLPNN  278

Athal_BKN3cDNA_Col-0  YKIGEIIDPRIGSDYPVDAATKIVTLIQRCTNRDKKKRPLMQEVLDALNYIAGIRS               196
AL6G22010.t1          YKIEEIIDPRLGSDYPVDAATKMFTLIQSCTKRDKKNRPLMQQVLDVLNNIAEIEY               334
Ahg10602              YKIEEIIDPRLGSDYPVDAATMMVTLIQSCTKRDRKNRPLMQQVLDVLNNIAGIKY               334
```

Fig. S5 *A. thaliana*, *A. lyrata and A. halleri* amino acid alignments for BKN1, BKN2 and BKN3.
a. BKN1 alignment, **b.** BKN2 alignment and **c.** BKN3 alignment.
Amino acid sequences were aligned using ClustalW in MEGA7.0 [101,106], and the alignments were formatted using Multiple Align Show [109]. Identical amino acids are shown in black text, similar amino acids are shown in blue text, and no identity are shown in red text. Percentage of sequences that must agree for identity or similarity coloring was set at 60%. Similar amino acids were grouped as: GAVLI (aliphatic), FYW (aromatic), CM (sulfur-containing), ST (hydroxyl), KRH (basic), DENQ (acidic and their amide), and P (cyclic) [110]. *A. lyrata* BKN1, 2 and 3 annotations were retrieved from Phytozome [102]. *A. halleri* BKN1 and BKN2 annotations were retrieved from EnsemblPlants [104], and *A. halleri* BKN3 annotation was retrieved from Phytozome (Araha.9474s0004.1). Both *A. halleri* BKN2 and BKN3 annotations were corrected based on *A. lyrata* exon/intron boundaries.

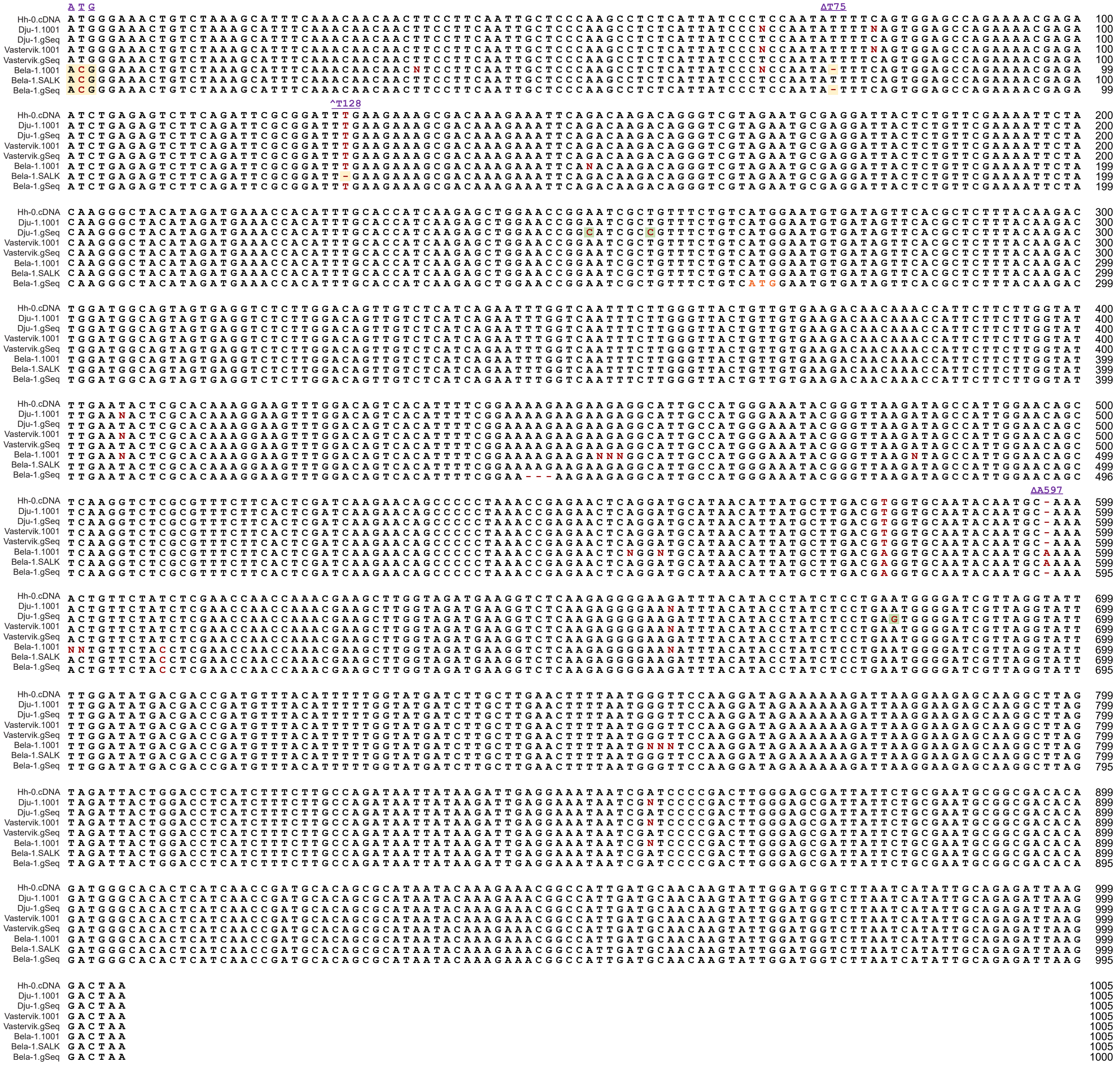

Fig. S6 Alignment of BKN1 coding sequences from four *A. thaliana* ecotypes.
From searches on the 1135 ecotype genome sequences, three ecotypes were predicted to carry two SNPs that would restore the *BKN1* reading frame (^T128, ΔA597): Hh-0, Västervik, Dju-1 (see Fig. S2 and Table S2 for more information). The Hh-0, Västervik, Dju-1 *BKN1* genes were all confirmed to encode full-length and identical proteins. The *BKN1* DNA sequences for Hh-0 and Västervik were 100% identical while the Dju-1 sequence displayed three synonymous SNPs (highlighted in green). Bela-1 was also predicted to carry the ^T128 and ΔA597 SNPs, but had an additional SNP mutating the start methionine (highlighted in yellow). The BKN1 coding regions were cloned and sequenced from these four ecotypes to confirm the predictions. The BKN1 cDNA was cloned from flower bud RNA for Hh-0, and genomic DNA samples were used to PCR amplify the BKN1 gene for Västervik, Dju-1 and Bela-1 (labeled .gSeq in the alignment). The exon only regions of these sequences were aligned, along with the sequences retrieved from two different databases: 1001 Genomes (https://1001genomes.org/) and Salk Arabidopsis 1,001 Genomes (http://signal.salk.edu/atg1001/index.php).

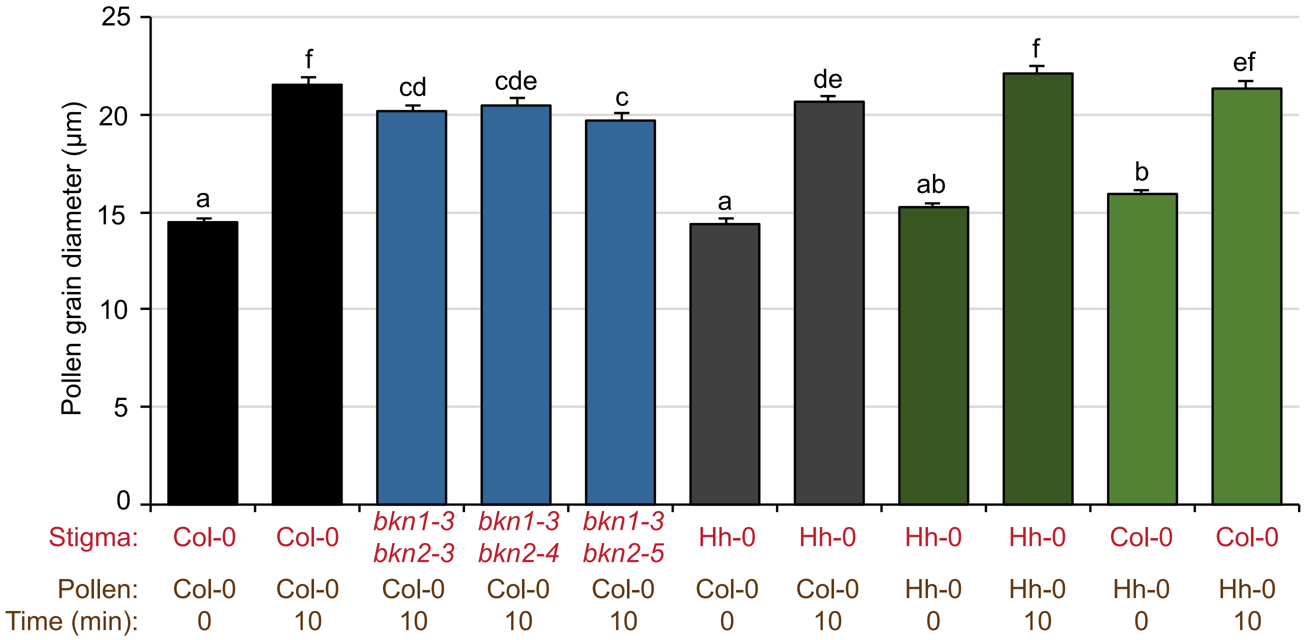

Fig. S7 Pollen Hydration Assays in the Col-0 and Hh-0 ecotypes at 10 minutes post-pollination. Different pollen-stigma combinations were tested: these were wild-type Col-0 pollen grains applied to Col-0 stigmas, the double *bkn1-bkn2* mutant stigmas (*bkn1-3 bkn2-3*, *bkn1-3 bkn2-4*, and *bkn1-3 bkn2-5*), and Hh-0 stigmas. As well, wild-type Hh-0 pollen grains were applied to Hh-0 stigmas and Col-0 stigmas.

Pollen hydration results in a change in pollen grain diameter which was measured at 0 min and 10 min post-pollination. n = 30 pollen grains per line.

Letters represent statistically significant groupings of $p<0.05$ based on a one-way ANOVA with a Duncan post-hoc test. Small but statistically significant decreases in wild-type Col-0 pollen hydration were observed for the double *bkn1-bkn2* mutant stigmas and the wild-type Hh-0 stigmas.

Fig. S8 Phylogenetic analysis of the Arabidopsis RLCK subfamily VII members.

A. thaliana RLCK VII members were defined by Lehti-Shiu and Shiu [51], and amino acid sequences were retrieved from TAIR [48]. Gene names were added from TAIR annotations, and the PBL names are from Rao et al [53]. The analysis involved 51 amino acid sequences and the sequences were aligned using ClustalW [106] in the MEGA 7 software [101]. The N-and C-terminal ends of the alignment were trimmed (see Supplemental files for sequences and alignment), and the tree was constructed using the Maximum Likelihood method [107] in the MEGA 7 software. All positions containing gaps and missing data were eliminated, and a total of 134 positions was in the final dataset. The tree generated in MEGA 7 represents the bootstrap consensus tree inferred from 1000 replicates [108]. KIN10 (At3g01090.2, SnRK1 kinase) was chosen as an outgroup.

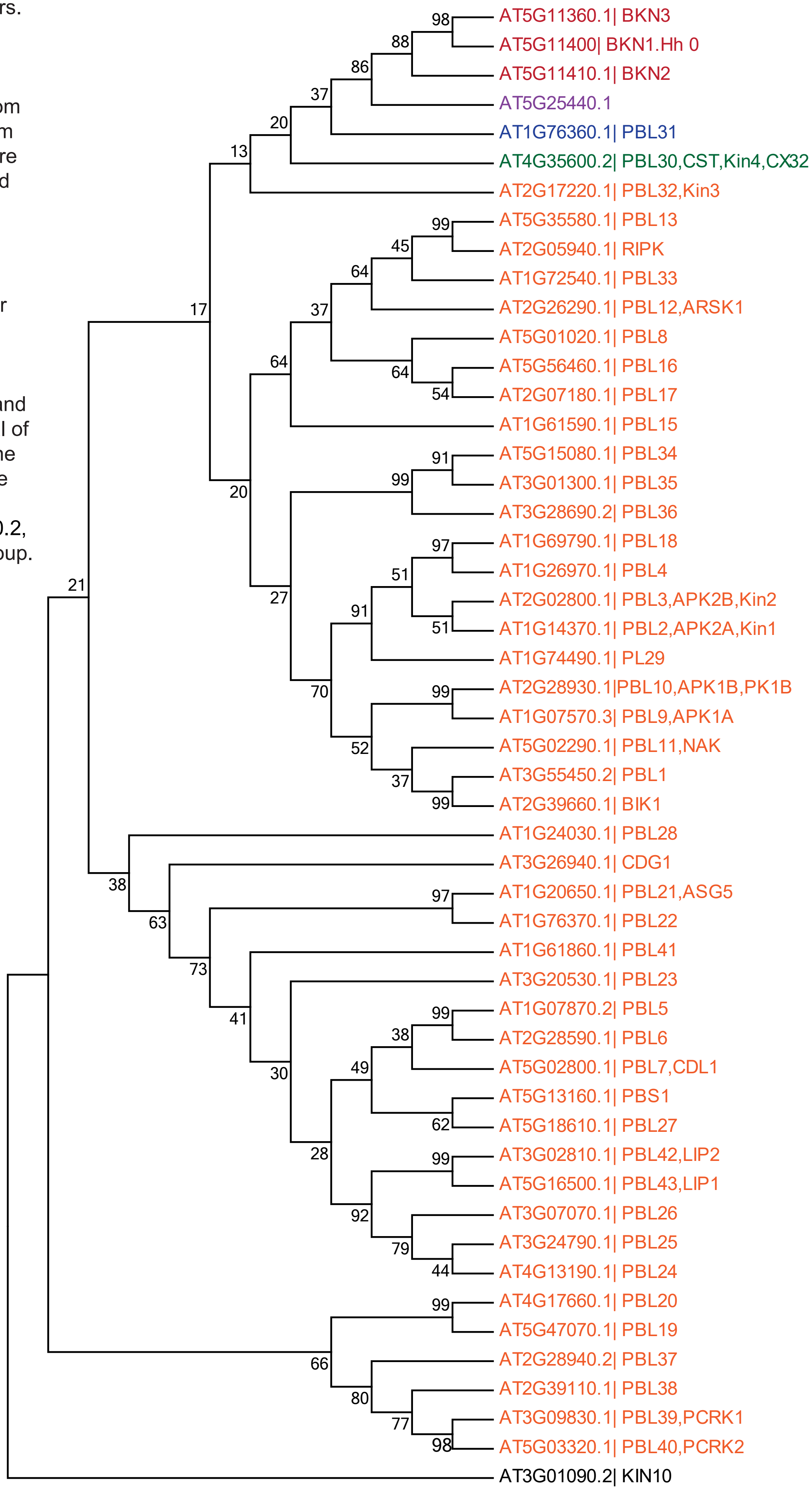

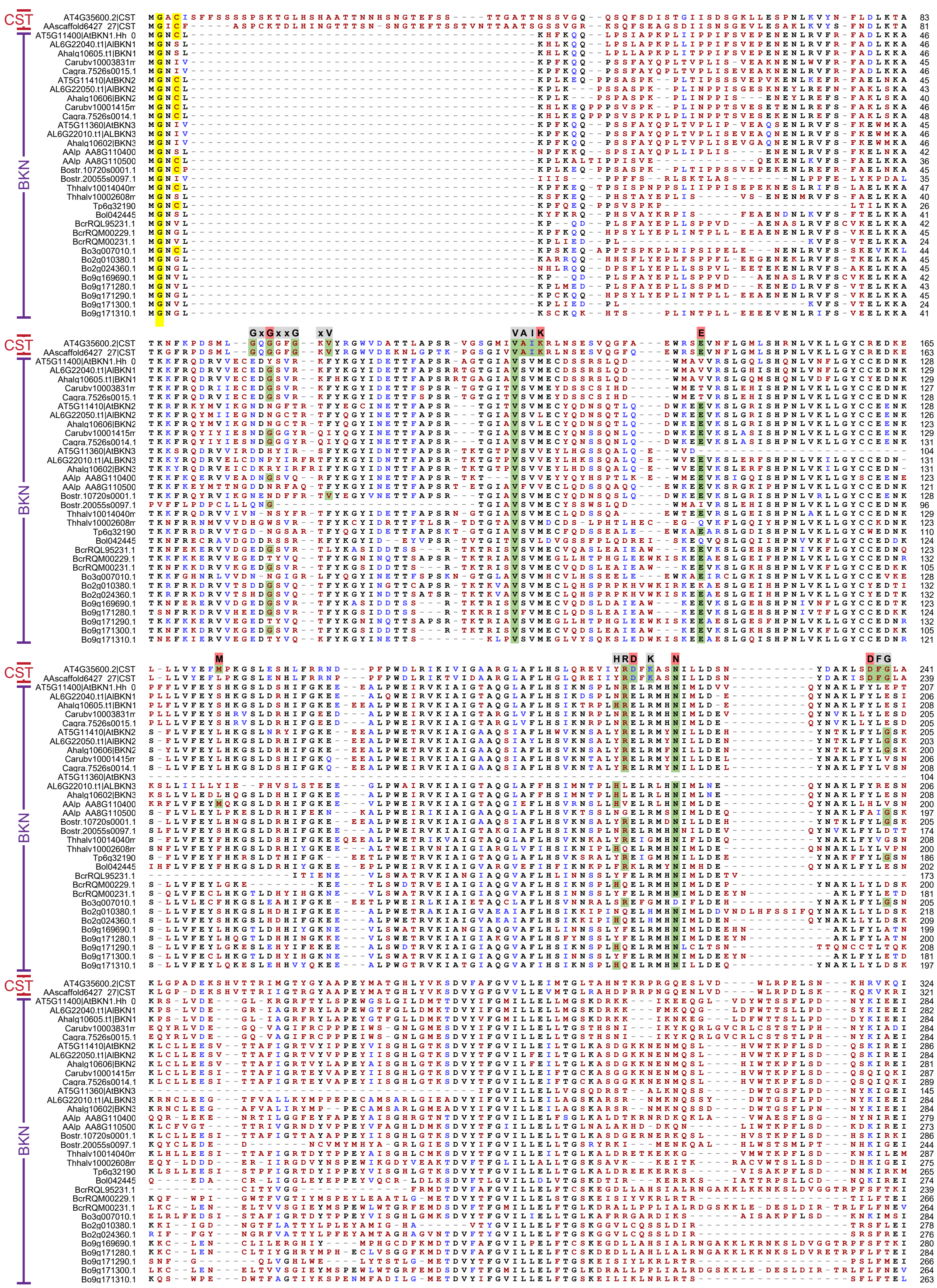

Fig. S9 Amino acid sequence alignment of BRASSIKIN (BKN) sequences with CASTAWAY (CST) sequences.
BKNs: *Arabidopsis lyrata* (AL), *A. thaliana* (AT), *A. halleri* (Ahal), *Capsella rubella* (Carub), *C. grandiflora* (Cagra), *Eutrema salsugineum* (Thhalv), *Schrenkiella parvula* (Tp), *Arabis alpina* (AApl), *Brassica cretica* (Bcr), *B. oleacea* (Bo), Boechera stricta (Bostr).
CSTs: *A. thaliana* (AT), *Aethionema arabicum* (AA).
Sequences were aligned using ClustalW in in MEGA7.0 [101,106], and the alignment was formatted using Multiple Align Show [109]. Identical amino acids are shown in black text, similar amino acids are shown in blue text, and no identity are shown in red text. Percentage of sequences that must agree for identity or similarity coloring was set at 60%. Similar amino acids were grouped as: GAVLI (aliphatic), FYW (aromatic), CM (sulfur-containing), ST (hydroxyl), KRH (basic), DENQ (acidic & their amides), and P (cyclic) [110]. Potential G2 myristoylation and C4 palmitoylation sites are highlighted in yellow.
Above the alignments are the consensus kinase motifs. The invariant kinase residues that contribute to ATP binding or catalytic acitivity are highlighted in red. Conserved residues [46] are highlighted in green. Based on the consensus motifs, CSTs are predicted to be active while BKNs are predicted to be pseudokinases.

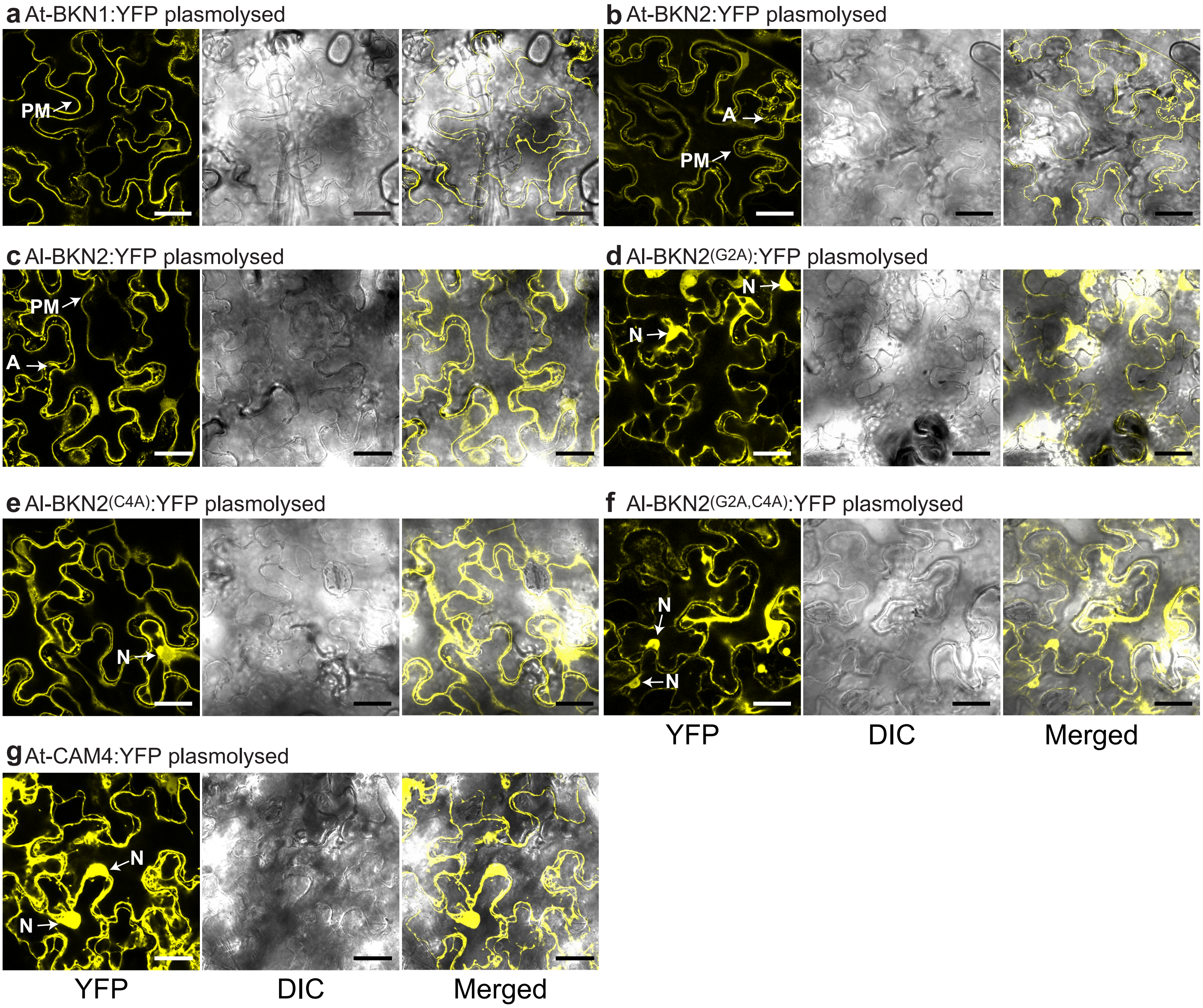

Fig. S10 Confocal microscopy imaging of *N. benthamiana* leaves infiltrated with C-terminal BKN:YFP fusion protein constructs and plasmolysed with 0.8M mannitol.
a-g. At-BKN1:YFP (Hh-0), At-BKN2:YFP, Al-BKN2:YFP, Al-BKN2$^{(G2A)}$:YFP, Al-BKN2$^{(C4A)}$:YFP, Al-BKN2$^{(G2A,C4A)}$:YFP, At-CAM4:YFP.
BKN1/2 localization in plasmolysed cells remains predominantly at the plasma membrane, with some localization to Hechtian strands in the apoplastic space (A). Hechtian strands are plasma membrane-cell wall connections [113]. With the Al-BKN2 versions mutated at the myristoylation (G2A) and/or palmitoylation (C4A) sites, the YFP signal is more diffuse and some localization to the nucleus was also observed.
PM = plasma membrane. *A. thaliana* CAM4:YFP was used to compare the localization of YFP fluorescence in the nucleus (N) and cytoplasm.
YFP fusion constructs were infiltrated at OD600=0.5, and images were taken 24-48 hours post-inoculation.
Scale bars = 30μm
